# Brain death and postmortem organ donation: report of a questionnaire from the CENTER-TBI study

**DOI:** 10.1186/s13054-018-2241-4

**Published:** 2018-11-16

**Authors:** Ernest van Veen, Mathieu van der Jagt, Maryse C. Cnossen, Andrew I. R. Maas, Inez D. de Beaufort, David K. Menon, Giuseppe Citerio, Nino Stocchetti, Wim J. R. Rietdijk, Jeroen T. J. M. van Dijck, Erwin J. O. Kompanje, CENTER-TBI investigators and participants, Cecilia Ackerlund, Hadie Adams, Vanni Agnoletti, Judith Allanson, Krisztina Amrein, Norberto Andaluz, Nada Andelic, Lasse Andreassen, Azasevac Antun, Audny Anke, Anna Antoni, Hilko Ardon, Gérard Audibert, Kaspars Auslands, Philippe Azouvi, Maria Luisa Azzolini, Camelia Baciu, Rafael Badenes, Ronald Bartels, Pál Barzó, Ursula Bauerfeind, Romuald Beauvais, Ronny Beer, Javier Belda Francisco, Bo-Michael Bellander, Antonio Belli, Rémy Bellier, Habib Benali, Thierry Benard, Maurizio Berardino, Luigi Beretta, Christopher Beynon, Federico Bilotta, Harald Binder, Erta Biqiri, Morten Blaabjerg, Hugo den Boogert, Pierre Bouzat, Peter Bragge, Alexandra Brazinova, Vibeke Brinck, Joanne Brooker, Camilla Brorsson, Andras Buki, Monika Bullinger, Emiliana Calappi, Maria Rosa Calvi, Peter Cameron, Guillermo Carbayo Lozano, Marco Carbonara, Elsa Carise, K. Carpenter, Ana M. Castaño-León, Francesco Causin, Giorgio Chevallard, Arturo Chieregato, Giuseppe Citerio, Maryse Cnossen, Mark Coburn, Jonathan Coles, Lizzie Coles-Kemp, Johnny Collett, Jamie D. Cooper, Marta Correia, Amra Covic, Nicola Curry, Endre Czeiter, Marek Czosnyka, Claire Dahyot-Fizelier, François Damas, Pierre Damas, Helen Dawes, Véronique De Keyser, Francesco Della Corte, Bart Depreitere, C. W. de Ruiter Godard, Dula Dilvesi, Shenghao Ding, Diederik Dippel, Abhishek Dixit, Emma Donoghue, Jens Dreier, Guy-Loup Dulière, George Eapen, Heiko Engemann, Ari Ercole, Patrick Esser, Erzsébet Ezer, Martin Fabricius, Valery L. Feigin, Junfeng Feng, Kelly Foks, Francesca Fossi, Gilles Francony, Ulderico Freo, Shirin Frisvold, Alex Furmanov, Pablo Gagliardo, Damien Galanaud, Dashiell Gantner, Guoyi Gao, Karin Geleijns, Pradeep George, Alexandre Ghuysen, Lelde Giga, Benoit Giraud, Ben Glocker, Jagos Golubovic, Pedro A. Gomez, Francesca Grossi, Russell L. Gruen, Deepak Gupta, Juanita A. Haagsma, Iain Haitsma, Jed A. Hartings, Raimund Helbok, Eirik Helseth, Daniel Hertle, Astrid Hoedemaekers, Stefan Hoefer, Lindsay Horton, Jilske Huijben, Peter J. Hutchinson, Asta Kristine Håberg, Bram Jacobs, Stefan Jankowski, Mike Jarrett, Bojan Jelaca, Ji-yao Jiang, Kelly Jones, Konstantinos Kamnitsas, Mladen Karan, Ari Katila, Maija Kaukonen, Thomas Kerforne, Riku Kivisaari, Angelos G. Kolias, Bálint Kolumbán, Erwin Kompanje, Ksenija Kolundzija, Daniel Kondziella, Lars-Owe Koskinen, Noémi Kovács, Alfonso Lagares, Linda Lanyon, Steven Laureys, Fiona Lecky, Christian Ledig, Rolf Lefering, Valerie Legrand, Jin Lei, Leon Levi, Roger Lightfoot, Hester Lingsma, Dirk Loeckx, Angels Lozano, Andrew I. R. Maas, Stephen MacDonald, Marc Maegele, Marek Majdan, Sebastian Major, Alex Manara, Geoffrey Manley, Martin Didier, Leon Francisco Martin, Costanza Martino, Armando Maruenda, Hugues Maréchal, Alessandro Masala, Julia Mattern, Charles McFadyen, Catherine McMahon, Béla Melegh, David Menon, Tomas Menovsky, Cristina Morganti-Kossmann, Davide Mulazzi, Visakh Muraleedharan, Lynnette Murray, Holger Mühlan, Nandesh Nair, Ancuta Negru, David Nelson, Virginia Newcombe, Daan Nieboer, Quentin Noirhomme, József Nyirádi, Mauro Oddo, Annemarie Oldenbeuving, Matej Oresic, Fabrizio Ortolano, Aarno Palotie, Paul M. Parizel, Adriana Patruno, Jean-François Payen, Natascha Perera, Vincent Perlbarg, Paolo Persona, Wilco Peul, Anna Piippo-Karjalainen, Sébastien Pili Floury, Matti Pirinen, Horia Ples, Maria Antonia Poca, Suzanne Polinder, Inigo Pomposo, Jussi Posti, Louis Puybasset, Andreea Radoi, Arminas Ragauskas, Rahul Raj, Malinka Rambadagalla, Ruben Real, Veronika Rehorčíková, Jonathan Rhodes, Samuli Ripatti, Saulius Rocka, Cecilie Roe, Olav Roise, Gerwin Roks, Jonathan Rosand, Jeffrey Rosenfeld, Christina Rosenlund, Guy Rosenthal, Rolf Rossaint, Sandra Rossi, Daniel Rueckert, Martin Rusnák, Marco Sacchi, Barbara Sahakian, Juan Sahuquillo, Oliver Sakowitz, Francesca Sala, Renan Sanchez-Porras, Janos Sandor, Edgar Santos, Luminita Sasu, Davide Savo, Nadine Schäffer, Inger Schipper, Barbara Schlößer, Silke Schmidt, Herbert Schoechl, Guus Schoonman, Rico Frederik Schou, Elisabeth Schwendenwein, Michael Schöll, Özcan Sir, Toril Skandsen, Lidwien Smakman, Dirk Smeets, Peter Smielewski, Abayomi Sorinola, Emmanuel Stamatakis, Simon Stanworth, Nicole Steinbüchel, Ana Stevanovic, Robert Stevens, William Stewart, Ewout W. Steyerberg, Nino Stocchetti, Nina Sundström, Anneliese Synnot, Fabio Silvio Taccone, Riikka Takala, Viktória Tamás, Päivi Tanskanen, Mark Steven Taylor, Braden Te Ao, Olli Tenovuo, Ralph Telgmann, Guido Teodorani, Alice Theadom, Matt Thomas, Dick Tibboel, Christos Tolias, Jean-Flory Luaba Tshibanda, Tony Trapani, Cristina Maria Tudora, Peter Vajkoczy, Shirley Vallance, Egils Valeinis, Gregory Van der Steen, Mathieu van der Jagt, Joukje van der Naalt, Jeroen T. J. M. van Dijck, Thomas A. van Essen, Wim Van Hecke, Caroline van Heugten, Dominique Van Praag, Thijs Vande Vyvere, Julia Van Waesberghe, Audrey Vanhaudenhuyse, Alessia Vargiolu, Emmanuel Vega, Kimberley Velt, Jan Verheyden, Paul M. Vespa, Anne Vik, Rimantas Vilcinis, Giacinta Vizzino, Carmen Vleggeert-Lankamp, Victor Volovici, Daphne Voormolen, Peter Vulekovic, Zoltán Vámos, Derick Wade, Kevin K. W. Wang, Lei Wang, Lars Wessels, Eno Wildschut, Guy Williams, Lindsay Wilson, Maren K. L. Winkler, Stefan Wolf, Peter Ylén, Alexander Younsi, Menashe Zaaroor, Yang Zhihui, Agate Ziverte, Fabrizio Zumbo

**Affiliations:** 1 Department of Intensive Care, Erasmus University Medical Center, Rotterdam, the Netherlands; 2 Center for Medical Decision Making, Department of Public Health, Erasmus University Medical Center, Rotterdam, the Netherlands; 3 Department of Medical Ethics and Philosophy of Medicine, Erasmus University Medical Center, Rotterdam, the Netherlands; 4 Department of Neurosurgery, Antwerp University Hospital and University of Antwerp, Edegem, Belgium; 5 Department of Anaesthesia, University of Cambridge, Cambridge, UK; 6 School of Medicine and Surgery, University of Milan-Bicocca, Milan, Italy; 7 San Gerardo Hospital, ASST-Monza, Monza, Italy; 8 Department of Physiopathology and Transplantation, Milan University, Milan, Italy; 9 Neuro ICU Fondazione IRCCS Cà Granda Ospedale Maggiore Policlinico Milano, Milan, Italy; 10 Department of Neurosurgery, Leiden University Medical Center, Leiden, the Netherlands

**Keywords:** Traumatic brain injury, Brain death, Ethics, Postmortem organ donation, Withdrawing life-sustaining measures, Ventricular drainage

## Abstract

**Background:**

We aimed to investigate the extent of the agreement on practices around brain death and postmortem organ donation.

**Methods:**

Investigators from 67 Collaborative European NeuroTrauma Effectiveness Research in Traumatic Brain Injury (CENTER-TBI) study centers completed several questionnaires (response rate: 99%).

**Results:**

Regarding practices around brain death, we found agreement on the clinical evaluation (prerequisites and neurological assessment) for brain death determination (BDD) in 100% of the centers. However, ancillary tests were required for BDD in 64% of the centers. BDD for nondonor patients was deemed mandatory in 18% of the centers before withdrawing life-sustaining measures (LSM). Also, practices around postmortem organ donation varied*.* Organ donation after circulatory arrest was forbidden in 45% of the centers. When withdrawal of LSM was contemplated, in 67% of centers the patients with a ventricular drain in situ had this removed, either sometimes or all of the time.

**Conclusions:**

This study showed both agreement and some regional differences regarding practices around brain death and postmortem organ donation. We hope our results help quantify and understand potential differences, and provide impetus for current dialogs toward further harmonization of practices around brain death and postmortem organ donation.

**Electronic supplementary material:**

The online version of this article (10.1186/s13054-018-2241-4) contains supplementary material, which is available to authorized users.

## Background

Before the 1950s, death was only determined using cardiovascular criteria. Due to advances in critical care medicine, especially mechanical ventilation, a new clinical state was observed in 1958 (i.e., “coma dépassé”) [1]. Although the systemic circulation was intact, the brain showed no objective evidence of function. This observation gave rise to the question of what “coma dépassé” meant. The successful transplantation of kidneys from a “coma dépassé” patient (1965) subsequently led to the first accepted standard for the confirmation of brain death in 1968 [2]. In 1981, the Uniform Determination of Death Act made death determined by neurological and cardiovascular criteria equivalent [3]. The American Academy of Neurology (AAN) in 1995 published guidelines for brain death determination (BDD) [4], and updated these in 2010 [5]. In 2008, the Academy of Medical Royal Colleges in the United Kingdom (UK) provided broader guidance on the determination of death in a range of circumstances, including BDD [6].

Brain death and postmortem organ donation are closely linked. Also, an important, and not well investigated, issue regarding circulatory arrest organ donation is the hands-off time after circulatory arrest. Practices around all of these mentioned topics are delicate. Thus, inconsistencies between centers can be confusing for the general public, and could expose clinicians to accusations of unethical practice. Consensus regarding practices around brain death and postmortem organ donation could prevent these inconsistencies. To facilitate this consensus, the first step is to document potential differences.

The Collaborative European NeuroTrauma Effectiveness Research in Traumatic Brain Injury (CENTER-TBI, www.center-tbi.eu) study addressed this issue. The CENTER-TBI study used questionnaires to create “provider profiles” of participating neurotrauma centers. One of these questionnaires intended to address specific practices around brain death and postmortem organ donation that currently provoke international discussion. Using this questionnaire, we aimed to quantify and understand potential differences, and provide impetus for current dialogs toward further harmonization of practices around brain death and postmortem organ donation. Regarding brain death, we investigated: criteria used for BDD; and the necessity of BDD before withdrawing life-sustaining measures (LSM). As for postmortem organ donation, we investigated: removal of the ventricular drain while continuing other LSM; the possibility for circulatory arrest organ donation; and the hands-off time after circulatory arrest.

## Methods

### CENTER-TBI and study sample

The CENTER-TBI study includes a prospective observational study on traumatic brain injury (TBI) [7, 8]. The investigators connected to this study collect data on patient characteristics, management, and outcomes in important centers from 20 countries across Europe and Israel. Investigators from all participating centers in the CENTER-TBI study were asked to complete several questionnaires. Centers were located in Austria (*N* = 2), Belgium (*N* = 4), Bosnia and Herzegovina (*N* = 2), Denmark (*N* = 2), Finland (*N* = 2), France (*N* = 7), Germany (*N* = 4), Hungary (*N* = 2), Israel (*N* = 2), Italy (*N* = 8), Latvia (*N* = 3), Lithuania (*N* = 2), the Netherlands (*N* = 7), Norway (*N* = 3), Romania (*N* = 1), Serbia (*N* = 1), Spain (*N* = 4), Sweden (*N* = 2), Switzerland (*N* = 1), and the UK (*N* = 8).

### Questionnaire development and administration

More detailed information about the development, administration, and content of the questionnaires is available from an earlier publication by Cnossen et al. [9].

The topics covered in the current study are summarized in Table 1. A complete overview of the questionnaires for this study can be found in Additional file 1: Questionnaire 1 (questions 1, 4, 8, and 9), 7 (questions 2 and 4), and 8 (questions 9 and 11–15). In the questionnaires, we explicitly asked for the “general policy” according to the investigators. We defined this as the local standards used in more than 75% of patients, recognizing that there might be exceptions. Most questions made use of categorical answer categories. For some questions, the investigators had the option to fill in an answer that could be different from one of the options provided. These answers were marked as “other” and consisted of free text responses. Where these free text responses from different investigators were sufficiently similar, we sought to combine them to provide additional categorical responses. We did this to facilitate summary descriptive statistics.

**Table 1:** Topics covered, related questions for each topic, and response rate per question

Topics covered in this study	Questions related to this topic	Response rate, *N* (%)
Practices around brain death		
Criteria for BDD	When do you declare a patient brain dead?	67 (99%)
Brain death and withdrawal of LSM	Must the patient, who is not suitable for organ donation, be declared brain dead before withdrawing life-sustaining measures?	67 (99%)
Practices around postmortem organ donation		
Donation after circulatory death	Would you consider organ donation after circulatory arrest in a patient in whom mechanical ventilation will be withdrawn, but who is not brain dead?	66 (97%)
Ventricular drain removal and organ donation	If the decision is made to withdraw life-sustaining measures, in a patient with high intracranial pressure, but who is not brain dead, would you remove the ventricular drain (for CSF drainage), but continue other life-sustaining measures in the hope that the patient will become brain dead and thereby becomes a suitable candidate for organ donation?	67 (99%)
Declaration of death and hands-off time in donors and nondonors	After withdrawal of mechanical ventilation and after circulatory arrest, when exactly do you declare the patient dead in case of a circulatory death organ donor?	64 (94%)
	After withdrawal of mechanical ventilation and after circulatory arrest, after how many minutes circulatory arrest do you declare the patient dead in cases not suitable as organ donor?	66 (97%)

*BDD* brain death determination, *CSF* cerebrospinal fluid, *LSM* life-sustaining measures

### Analyses

We used descriptive statistics to describe our outcomes. We calculated frequencies and percentages for all variables related to the number of responses for that question. Centers at which the investigator did not respond to every question remained in our study, in order to keep groups for descriptive statistics as large as possible. The response rates per question are presented in Table 1. We grouped countries into seven regions: Baltic States (Latvia and Lithuania), Eastern Europe (Bosnia and Herzegovina, Hungary, Romania, and Serbia), Israel, Northern Europe (Denmark, Finland, Norway, and Sweden), Southern Europe (Italy and Spain), the United Kingdom, and Western Europe (Austria, Belgium, France, Germany, the Netherlands, and Switzerland). We examined potential differences between and within regions.

## Results

### Center characteristics

Of the 68 centers, investigators from 67 centers participated in the questionnaires (response rate: 99%) and were included in the analysis. The participating centers were mainly academic centers (*N* = 61, 91%), designated as a level I or II trauma center (*N* = 49, 73%). The average number of beds in the participating centers was 1187, of which on average 39 were intensive care unit (ICU) beds. The average number of annual treatments per ICU in 2013 was 1408, of which on average 130 were TBI patients.

### Practices around brain death

#### When do you declare a patient brain dead?

We found agreement on the clinical evaluation (prerequisites and neurological assessment) for BDD in 100% of the centers. The clinical evaluation for BDD included: a Glasgow Coma Scale (GCS) of three, absence of brain stem reflexes, no respiratory efforts in response to an apnea test, and absence of confounding factors to evaluate consciousness (e.g., hypothermia). However, ancillary tests were required for BDD in 43 (64%) centers (Table 2).

**Table 2:** Practices around brain death

	Region							
Answer	Sample total (*N* = 67)	Baltic States (*N* = 5)	Eastern Europe (*N* = 6)	Israel (*N* = 2)	Northern Europe (*N* = 9)	Southern Europe (*N* = 12)	United Kingdom (*N* = 8)	Western Europe (*N* = 25)
When do you declare a patient brain dead?								
With GCS 3, fixed dilated pupils, and no confounding factors (e.g., hypothermia, barbiturates)	0	0	0	0	0	0	0	0
With GCS 3 and absent brain stem reflexes, and no confounding factors	0	0	0	0	0	0	0	0
With GCS 3, absent brain stem reflexes and apnea, and no confounding factors	31	20	17	0	78	0	88	20
With GCS 3, absent brain stem reflexes, apnea and ancillary test(s) (e.g., EEG or cerebral angiography), and absence of confounding factors	64	80	83	100	22	100	0	72
Per national protocol^a^	4	0	0	0	0	0	13	8
Must the patient, who is not suitable for organ donation, be declared brain dead before withdrawing LSM?								
No, the prospect of a very poor prognosis can be enough	61	0	17	0	78	42	100	80
No, GCS 3 and fixed dilated pupils and no confounders is enough to stop treatment	13	0	0	50	22	8	0	20
Yes, this is mandatory by law in my country	18	80	17	50	0	50	0	0
Yes, it is not mandatory by law, but I always do that to be sure	7	20	67	0	0	0	0	0

Data presented as percentage

*EEG* electroencephalography, *GCS* Glasgow Coma Scale, *LSM* life-sustaining measures

^a^Additional categorical responses, while free text responses were sufficiently similar. This does not mean that the other centers do not follow their national protocol

In three regions (43%; Israel, Southern Europe, and the UK), the same criteria for BDD were used in every center of the same region. In centers from Northern Europe and the UK, ancillary tests were rarely used for BDD (*N* = 2, 22% and *N* = 0, 0%, respectively).

#### Must the patient, who is not suitable for organ donation, be declared brain dead before withdrawing LSM?

The declaration of brain death in nondonor patients was mandatory before withdrawing LSM in 12 (18%) centers. In 41 (61%) centers, a poor prognosis as assessed by the treating physician(s) was considered sufficient. In 9 (13%) centers, a GCS score of three, fixed dilated pupils, and absence of confounders could motivate withdrawing LSM (Table 2).

In all centers in the Baltic States (*N* = 5), nondonor patients were declared brain dead before withdrawing LSM. In several centers in Eastern Europe and Southern Europe (*N* = 1, 17% and *N* = 6, 50%, respectively), it was mandatory to declare a patient brain dead before withdrawing LSM in nondonor patients, whereas in other centers from the same region this was not mandatory.

### Practices around postmortem organ donation

#### Would you consider organ donation after circulatory arrest in a patient in whom mechanical ventilation will be withdrawn, but who is not brain dead?

**Fig. 1:**
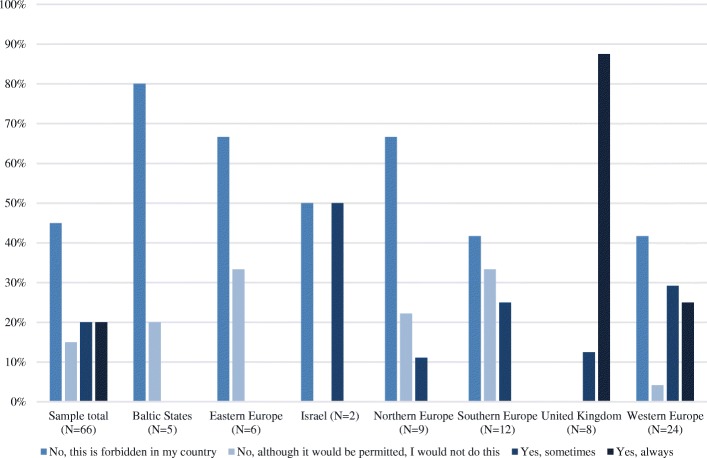
Results of question 13 (Questionnaire 8): Would you consider organ donation after circulatory arrest in a patient in whom mechanical ventilation will be withdrawn, but who is not brain dead?

Organ donation after circulatory arrest was forbidden in 30 (45%) centers (Fig. 1 and Table 3).

**Table 3:** Practices around circulatory arrest organ donation and ventricular drain removal

	Region							
Answer	Sample total (*N* = 66)	Baltic States (*N* = 5)	Eastern Europe (*N* = 6)	Israel (*N* = 2)	Northern Europe (*N* = 9)	Southern Europe (*N* = 12)	United Kingdom (*N* = 8)	Western Europe (*N* = 24)
Would you consider organ donation after circulatory arrest in a patient in whom mechanical ventilation will be withdrawn, but who is not brain dead?								
No, this is forbidden in my country	45	80	67	50	67	42	0	42
No, although it would be permitted, I would not do this	15	20	33	0	22	33	0	4
Yes, sometimes	20	0	0	50	11	25	13	29
Yes, always	20	0	0	0	0	0	88	25
	Sample total (*N* = 67)	Baltic States (*N* = 5)	Eastern Europe (*N* = 6)	Israel (*N* = 2)	Northern Europe (*N* = 9)	Southern Europe (*N* = 12)	United Kingdom (*N* = 8)	Western Europe (*N* = 25)
If the decision is made to withdraw life-sustaining measures, in a patient with high intracranial pressure, but who is not brain dead, would you remove the ventricular drain (for CSF drainage), but continue other life-sustaining measures in the hope that the patient will become brain dead and then becomes a suitable candidate for organ donation?								
No, never	33	80	33	0	0	17	88	28
Yes, sometimes	51	20	50	100	100	50	13	48
Yes, always	16	0	17	0	0	33	0	24

Data presented as percentage

*CSF* cerebrospinal fluid

In all centers in the UK (*N* = 8), postmortem organ donation after circulatory arrest was approved. In centers in the Baltic States, Eastern Europe, and Northern Europe, organ donation after circulatory arrestwas often forbidden (*N* = 4, 80%; *N* = 4, 67% and *N* = 6, 67% respectively).

#### If the decision is made to withdraw life-sustaining measures, in a patient with high intracranial pressure, but who is not brain dead, would you remove the ventricular drain (for CSF drainage), but continue other life-sustaining measures in the hope that the patient will become brain dead and thereby becomes a suitable candidate for organ donation?

**Fig. 2:**
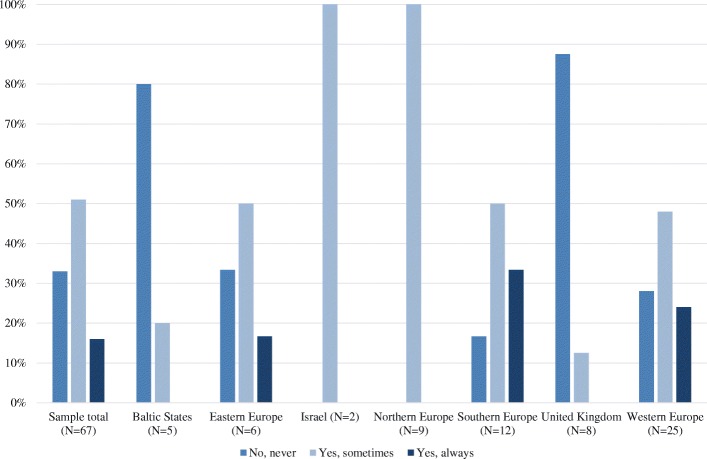
Results of question 9 (Questionnaire 8): If the decision is made to withdraw life-sustaining measures, in a patient with high intracranial pressure, but who is not brain dead, would you remove the ventricular drain (for CSF drainage), but continue other life-sustaining measures in the hope that the patient will become brain dead and thereby becomes a suitable candidate for organ donation?

In 45 (67%) centers, the ventricular drain was sometimes or always removed. In 11 of these 45 centers (16% of the Sample total), the ventricular drain was always removed while continuing other LSM. In 22 (33%) centers, the ventricular drain was never removed while continuing other LSM (Fig. 2 and Table 3).

In 4 (80%) centers in the Baltic States and in 7 (88%) centers in the UK, the ventricular drain was never removed. In all centers from Israel (*N* = 2) and Northern Europe (*N* = 9), the ventricular drain was “sometimes” removed.

#### After withdrawal of mechanical ventilation and after circulatory arrest, when exactly do you declare the patient dead in case of a circulatory death organ donor, and in cases not suitable as an organ donor?

In the case of a circulatory death organ donor, it was most common (*N* = 15, 23%) to declare the patient dead after 5-min “flatliner-ECG”. In cases not suitable as an organ donor, it was most common (*N* = 21, 32%) to declare the patient dead directly after detection of a “flatliner-ECG” on the monitor (Table 4).

**Table 4:** Practices around the hands-off time after circulatory arrest

	Region							
Answer	Sample total (*N* = 64)	Baltic States (*N* = 5)	Eastern Europe (*N* = 6)	Israel (*N* = 2)	Northern Europe (*N* = 9)	Southern Europe (*N* = 12)	United Kingdom (*N* = 8)	Western Europe (*N* = 22)
After withdrawal of mechanical ventilation and after circulatory arrest, when exactly do you declare the patient dead in case of a circulatory death organ donor?								
Directly after circulatory arrest determined after a “flatliner-ECG” on the monitor	16	40	0	50	11	8	0	23
After 1-min “flatliner-ECG” indicating circulatory arrest	5	0	0	50	0	8	0	5
After 2-min “flatliner-ECG”	2	0	0	0	0	0	0	5
After 5-min “flatliner-ECG”	23	20	33	0	11	17	50	23
After 10-min “flatliner-ECG”	5	20	17	0	0	0	0	5
After loss of pulsatile arterial curve on the invasive arterial blood pressure tracing	6	20	17	0	0	0	0	9
After 20-min “flatliner-ECG”^a^	11	0	0	0	0	58	0	0
Not done in our hospital/country^a^	19	0	17	0	78	0	0	18
Other, please specify^b^	14	0	17	0	0	8	50	14
	Sample total (*N* = 66)	Baltic States (*N* = 5)	Eastern Europe (*N* = 6)	Israel (*N* = 2)	Northern Europe (*N* = 9)	Southern Europe (*N* = 12)	United Kingdom (*N* = 8)	Western Europe (*N* = 24)
After withdrawal of mechanical ventilation and after circulatory arrest, after how many minutes circulatory arrest do you declare the patient dead in cases **not** suitable as organ donor?								
Directly after circulatory arrest determined after a “flatliner-ECG” on the monitor	32	40	17	100	11	17	13	50
After 1-min “flatliner-ECG” indicating circulatory arrest	5	0	0	0	0	0	0	13
After 2-min “flatliner-ECG”	0	0	0	0	0	0	0	0
After 5-min “flatliner-ECG”	23	20	17	0	22	25	38	21
After 10-min “flatliner-ECG”	6	20	33	0	0		0	0
After loss of pulsatile arterial curve on the invasive arterial blood pressure tracing	6	20	33	0	11	0	0	0
After 20-min “flatliner-ECG”^a^	9	0	0	0	0	50	0	0
Not done in our hospital/country^a^	8	0	0	0	33	0	0	8
Other, please specify^c^	12	0	0	0	22	0	50	8

Data presented as percentage

*EEG* electroencephalography

^a^Additional categorical responses, while free text responses were sufficiently similar

^b^Specifications filled in under “other”: “two minutes after loss of pulsatile arterial curve on the invasive arterial blood pressure tracing”; “after 3 min”; “No carotid pulses and apnoea”; “absence central pulse for 5 mins confirmed by observation for further 5 mins”; “National guidance 5 mins mechanical asystole”; “apnea test positivity”; “according to the Dutch law on organ donation”; “Protokollbogen zur Feststellung des irreversiblen Hirnfunktionsausfalls”; “at the beginning of the commission observation (6 h before)”

^c^Specifications filled in under “other”: “Control 10 min later”; “After clinical death diagnosis: listen to heart sound, examination of pupils”; “At decision of the physician”; “No carotid pulses and apnoea”; “absence central pulse for 5 mins confirmed by observation for further 5 mins”; “apnea test positivity”; “according to the Dutch law on organ donation”; “Protokollbogen zur Feststellung des irreversiblen Hirnfunktionsausfalls”; “at the beginning of the commission observation (6 h before)”

In all centers in Israel, nondonor patients were declared dead directly after detection of a “flatliner-ECG” on the monitor. No other region had the same answer in every center concerning the declaration of death in donor and nondonor patients.

## Discussion

We aimed to investigate specific practices that currently provoke international discussion in the area of brain death and postmortem organ donation. We aimed to quantify and understand potential differences, and provide impetus for current dialogs toward further harmonization of practices around brain death and postmortem organ donation.

Taking all results together, we found agreement on the clinical evaluation (prerequisites and neurological assessment) for brain death determination (BDD) across regions. In addition to this clinical evaluation, ancillary tests were required for BDD in 64% of the centers. BDD was deemed mandatory before withdrawal of life-sustaining measures (LSM) even outside the context of organ donation in 18% of the centers. As for practices around postmortem organ donation across regions, in 67% of the centers a ventricular drain was sometimes or always removed while other LSM were continued. Last, in 45% of the centers organ donation after circulatory arrest was forbidden.

We found important agreement and some differences regarding practices around brain death. Due to the broad categorical answer possibilities provided, the application of these findings is limited. First, agreement existed in all centers on the clinical evaluation for BDD, namely a Glasgow Coma Scale (GCS) of three, absence of brain stem reflexes, no respiratory efforts in response to an apnea test, and absence of confounding factors to evaluate consciousness. This is promising, in the light of recent calls to reach a worldwide consensus on how to determine brain death [10]. However, in addition to this clinical evaluation, ancillary tests were reported to be required for BDD in two thirds of centers. These differences in the use of ancillary tests are in line with previous literature [11–19]. Interestingly, however, there have been calls to abandon ancillary tests for BDD [20]. In the majority of centers from Northern Europe and the United Kingdom (UK), ancillary tests were not mandatory for BDD. This is in line with the study by Wahlster et al. [11]. These discrepancies may suggest differences in ethical principles and regulatory practice between centers. In some centers it was mandatory to declare nondonor patients brain dead before withdrawing life-sustaining measures (LSM). Withdrawal of LSM and the declaration of brain death are two different processes. The obligation of BDD before limiting treatment is debatable, since many non-brain dead patients may have a hopeless prognosis rendering further treatment futile.

We also found differences regarding practices around postmortem organ donation. First, we found differences concerning the removal of the ventricular drain. Our questionnaire did not assess in-depth the reasons why some centers opted to discontinue drainage and remove the ventricular drain as compared to maintaining the device in place, and how such continued intervention was incorporated into the care plan. Second, we found differences with regard to the possibility for organ donation after circulatory arrest. These results are in line with previous literature [21, 22]. The ventricular drain (mentioned earlier in this paragraph) seemed to be removed more often in centers where donation after circulatory arrest was not possible. If this turns out to be general practice, this might indicate the need for reevaluation of organ donation after circulatory arrest in order to prevent future burdensome care. For international figures on donation and transplantation, we refer the reader to the Newsletter Transplant 2017 produced by the Council of Europe of the European Committee [23]. There are no specific figures available for the centers involved in the Collaborative European NeuroTrauma Effectiveness Research in Traumatic Brain Injury (CENTER-TBI) study. Although the CENTER-TBI study includes important neurotrauma centers, we do not know to what extent these centers are responsible for the investigated figures of the Council of Europe. For the countries involved in our study, the number of donations after brain death in 2016 varied between 1.3 per million inhabitants (Bosnia and Herzegovina) and 33.1 per million inhabitants (Spain) [23]. Third, we found differences in hands-off times needed after circulatory arrest in order to declare a patient dead. This could indicate a lack of clear evidence on the exact time needed to be sure the brain has irreversibly lost its function.

Some of the differences appear region specific, but for other aspects we found variation between centers within a single region. Differences were even noted between regions participating in Eurotransplant, an organization that aims to optimally distribute organs by transplanting across national borders, when no matching recipient is available on the waiting list in the donor’s country. Eurotransplant covers part of Europe, and includes eight countries: Austria, Belgium, Croatia, Germany, Hungary, the Netherlands, Luxembourg, and Slovenia. The differences found pertained to all topics covered in this study.

Present-day medicine is said to be affected by the cultural climate of the society in which it exists [24]. This may indicate that differences in culture could explain some of the observed variation. Other results, such as possibilities for organ donation after circulatory arrest, suggest that variations have a more legal or regulatory basis. Observed within-region differences which suggest a more legal or regulatory basis raise questions regarding the level of enforcement of pertinent laws, and may indicate a lack of knowledge, regulatory implementation, or ambiguous legislation.

This study has several limitations that should be considered when interpreting the results. First, the participating neurotrauma centers represent a select group. The data obtained may therefore not be representative for all neurotrauma centers within the geographical areas studied. Second, our sample size made it difficult to apply more advanced statistics, such as a chi-square test, cluster analysis, and multidimensional scaling. Third, the results are based on the perceptions of practices reported by specific investigators rather than on clinical data. The CENTER-TBI study will further clarify actual practices around brain death and postmortem organ donation by analyzing clinical data. Fourth, investigators may have interpreted some questions incorrectly because a questionnaire does not always permit the nuances appropriate for clinical practice. In clinical practice, potential alternative options are both more numerous and complex than can be captured by a questionnaire. Last, investigators may have presented (even unwittingly) a more favorable image or presented individual preferences instead of the general policy in a center that we asked for.

Future research should focus on extending this study to a larger group of neurotrauma centers across the world in order to examine (in more advanced statistics) whether our results also apply to other centers. Furthermore, it would be interesting to study the origin of the differences found (e.g., cultural differences and differences pertaining to legislation). The complexity of some of the drivers of reported practice makes the case for mixed methods approaches to this problem, with a potentially substantive role for qualitative research methods. These strategies are important in order to inform preferred approaches to improve harmonization in neurotrauma centers across Europe and Israel.

Most importantly, current dialogs should be continued, and we hope that our findings may provide a basis toward further harmonization of practices around brain death and postmortem organ donation.

## Conclusion

This study showed both agreement and some regional differences regarding practices around brain death and postmortem organ donation. We hope our results help quantify and understand potential differences, and provide impetus for current dialogs toward further harmonization of practices around brain death and postmortem organ donation.

## Additional file

Additional file 1: Provider profiling questionnaires used for this study (Questionnaires 1, 7, and 8). (PDF 596 kb)

## Abbreviations

AAN: American Academy of Neurology
BDD: Brain death determination
CENTER-TBI: Collaborative European NeuroTrauma Effectiveness Research in Traumatic Brain Injury
CSF: Cerebrospinal fluid
ECG: Electrocardiography
GCS: Glasgow Coma Scale
ICU: Intensive care unit
LSM: Life-sustaining measures
TBI: Traumatic brain injury
UK: United Kingdom
