# Supplementary material for: Brain death and postmortem organ donation: report of a questionnaire from the CENTER-TBI study
Source: Crit Care. 2018 Nov 16;22:306. doi: 10.1186/s13054-018-2241-4 (PMC6240295; doi:10.1186/s13054-018-2241-4)
Supplement: Supplementary file 1 — Provider profiling questionnaires used for this study (Questionnaires 1, 7, and 8). (PDF 596 kb) [file 13054_2018_2241_MOESM1_ESM.pdf]

Provider Profiling Questionnaire

Questionnaire 1: General Questions

This questionnaire can be completed by the CENTER-TBI local investigator

The Local investigator is the senior clinician(s) at your hospital involved in supervision of CENTER TBI

For the completion of this questionnaire, we advise you to ask help from a data manager, administrative staff member and/or someone from the financial department in your hospital, since we ask for hospital data in this questionnaire. It is very important that this information is accurate, and searched for in annual reports, registries and other data sources rather than estimated.

This questionnaire also includes questions about the general policy in your hospital. The responses to these questions should represent, as best as practicable, a general consensus on treatment at your centre, rather than individual management preferences. Consequently, you should provide responses that describe not what you would do personally, but how the majority of patients would generally be treated in your centre.

There are no 'right' or 'wrong' answers so please give us a realistic and honest view of how the care in your hospital is organized. Your answers will only be used to answer the scientific questions in CENTER TBI and no information in any form will be reported on individual centre level. Some of the questions may seem similar, but please answer all questions.

If you have any questions or problem, please contact:
Maryse Cnossen, PhD student (m.c.cnossen@erasmusmc.nl)

Information about the completer of the questionnaire

Other than the CENTER-TBI investigator, which of the following individuals was involved in completion of this questionnaire?
Select all that apply

- ☐ Neurologist
- ☐ Neurosurgeon
- ☐ Trauma Surgeon
- ☐ Emergency Department (ED) physician
- ☐ Administrative staff member / data manager / financial department
- ☐ Other, please specify…………….
- ☐ NA. The questionnaire was completed solely by the CENTER TBI local investigator

Structural characteristics of the hospital

1.What type of hospital is your hospital?

- o Academic / University hospital
- o Nonacademic hospital

Academic / University Hospital = when your hospital is part of or affiliated to a University. An Academic/University hospital aims not only to deliver high-standard patient care, but also contributes to research and education

2. What is the hospital location?

- o Urban
- o Suburban
- o Rural

Urban location = a hospital in or very near to a city; the area is crowded<br>Suburban location = in between urban and rural location.<br>Rural location = a hospital in a location in or very near to the countryside, the area is not crowded

3. How are important wards / facilities in your hospital for Traumatic Brain Injury (TBI) patients (e.g. Emergency Department (ED), Intensive Care Unit (ICU), hospital ward, surgery rooms, scanning) situated?

- o All facilities that are related to TBI patients are in the same building and on same floor
- o Facilities are distributed over different buildings
- o Facilities are located in the same building but on different floors

Some hospitals consist of different buildings. Only tick the different buildings or different floor option if this affects TBI patients. So think of the typical course of a TBI patient (ED, ICU, hospital ward, surgery, CT scan, MRI scan etc.). Are these facilities situated in different buildings or on different floors?

4. Is your hospital officially designated as trauma centre?

- o No
- o Yes
- o N/A in our country/region

In some countries, hospitals designated as trauma centres are categorized, for example level 1 or 2 trauma centre. In other countries, these categories do not apply. If you live in a country that does not explicitly designate trauma centres, please select the N/A box.

4b. If your hospital is a designated Trauma Centre, what is the designation level?

- o Level I
- o Level II
- o Level III

A **Level I trauma centre** is a regional resource centre and generally serves large cities or population-dense areas. A level I trauma centre is expected to manage large numbers of severely injured patients (at least 1,200 trauma patients yearly or have 240 admissions with an Injury Severity Score of more than 14). It is characterized by 24-hour in-house availability of an attending surgeon and the prompt availability of other specialties (e.g. neurosurgeon, trauma surgeon).

A **Level II trauma centre** provides comprehensive trauma care in either a population-dense area in which a level II trauma centre may supplement the clinical activity and expertise of a level I institution or occur in less population-dense areas. In the latter case, the level II trauma centre serves as the lead trauma facility for a geographic area when a level I institution is not geographically close enough to do so. It is characterized by 24-hour in-house availability of an attending surgeon and the prompt availability of other specialties (e.g. neurosurgeon, trauma surgeon).

A **level III trauma centre** has the capacity to initially manage the majority of injured patients and have transfer agreements with a level I or II trauma center for seriously injured patients whose needs exceed the facility's resources. A level III trauma centre must have continuous general surgical coverage

4c. If your hospital is a designated Trauma Centre how far away is the next nearest designated trauma centre?

- ○ …………………..km
- ○ ……………………miles

5. What Glasgow Coma Scale (GCS) scores are considered as mild, moderate and severe Traumatic Brain Injury (TBI) in your hospital:

	Lowest GCS value	Highest GCS values
Mild TBI	……………………	……………………
Moderate TBI	……………………	……………………
Severe TBI	……………………	……………………

There are differences between countries and hospitals in how they classify mild, moderate and severe TBI. Please give the lowest and highest GCS values that is considered as mild, moderate and severe. For example: severe TBI might have a lowest value of 3 and a highest value of 8.

You can use hospital or national guidelines here. If these are not available, we would recommend, for example, an email exchange with colleagues to check that the answer that you provide us here represents the view of most of the persons in your department.

6. What specialty is most often responsible (in charge) for the treatment of Traumatic Brain Injury (TBI) patients in your hospital? Distinguish Emergency Department (ED), Hospital ward (ward where TBI patients are generally admitted) and Intensive Care Unit (ICU) stay?

Please specify per stratum, what specialism is most often in charge. This does not automatically have to be the person with the most contact hours with the patients.

Only select N/A if it is not possible to select one specialty that is most often in charge.

	ED	Ward	ICU
ED physician	☐	☐	☐
Neurologist	☐	☐	☐
Neurosurgeon	☐	☐	☐
Intensivist	☐	☐	☐
Trauma Surgeon	☐	☐	☐
General or Orthopaedic surgeon	☐	☐	☐
N/A. Care for TBI patients Is always	☐	☐	☐

multidisciplinary			
Other, please specify……………………….		☐	☐

Catchment Area

7. How many other hospitals within a 5 and 10 km radius of your hospital receive and treat patients with severe Traumatic Brain Injury (TBI)?
5 km ………… hospitals
10 km ………… hospitals

This question refers to other hospitals that have the capacity to treat severe TBI patients.<br>Exclude hospitals that receive severe TBI, but only stabilize and transfer patients to another hospital

Volume
8. How many beds does your hospital have in total?
……………………………………..

9. How many beds does your hospital have in the following wards and how many of these beds are potentially available for Traumatic Brain Injury (TBI) patients?

Ward	Total number of beds	Number of beds available for TBI patients
Emergency department	…………………………..	…………………………..
Trauma ward	…………………………..	…………………………..
Trauma surgery ward	…………………………..	…………………………..
Neurology ward	…………………………..	…………………………..
Neurosurgery ward	…………………………..	…………………………..
Intensive Care Unit	…………………………..	…………………………..
Surgical ward	…………………………..	…………………………..
Another ward where TBI patients may be referred to. Please specify……………..	…………………………..	…………………………..

Please provide the total amount of beds in your hospital per ward in the first column (total number of beds) and then search for the number of beds that are potentially available for TBI patients. This is the total number of beds minus the beds that are already scheduled for something else (for example planned operations).<br><br>If you do not have one of the mentioned wards, then answer 0 under total number of beds and number of beds available for TBI patients.<br><br>It might be possible that in your hospital, some of these wards are combined, for example a combination between the neurology and neurosurgery beds (clinical neuroscience). If that is the case, type 0 as the entry for the neurology and neurosurgery rows, and type: ‘clinical neuroscience’ under ‘another ward’. Please also explain briefly what your entry means: E.g. “clinical neuroscience = beds for neurology and neurosurgery patients”

Surgical facilities
10. How many operation rooms does your hospital have in total?
…………………………………………………….

This question refers to the total number of operation rooms in your hospital, not just the operating rooms that might be related to the care of TBI patients

11. How many operation rooms are potentially available for Traumatic Brain Injury (TBI) patients?

………………………………………………..

Here, we want to know how many rooms are available for TBI patients. You will first have to find out which operation rooms are used for surgery in TBI patients (trauma operating rooms, neurosurgical operating rooms etc.), and then count these numbers.<br>Exclude operating rooms that are used for non-TBI surgery in TBI patients – e.g. orthopaedic surgery in patients with multiple trauma

12. Does your hospital have separate 24/7 emergency operating rooms?
- o No
- o Yes

The response to this question should address operating rooms that are exclusively used for emergency surgery, and **never** used for planned or elective surgery

13. Are Operating Room (OR) personnel available 24/7?
Tick one option here
- o No.
- o Yes. There is 24/7 in-house availability of OR personnel
- o Yes. OR personnel are on call and would arrive in response to a call out within 30 minutes
- o Yes. OR personnel are on call and would arrive in response to a call out, but not within 30 minutes

The term "OR personnel" in this question refers to Operating Room nurses, anesthesiologists, anesthesiology assistants, etc.

Staffing

14. Do you have a residency program for the following clinical specialties in your hospital? If the answer is 'yes', how many years do these residency programs take, and how many trainees are appointed to the start of the program in <u>your</u> hospital?

Specialism:	Yes / No:	Number of years :	Number of trainees that start annually (maximum):
Trauma surgeon	Yes / No	….. years	………. trainees
Neurosurgeon	Yes / No	….. years	………. trainees
Neurologist	Yes / No	….. years	………. trainees
Intensivist	Yes / No	….. years	………. trainees

In this response, the term 'trauma surgeon' refers to an individual who specializes in trauma surgery, not a general surgeon or orthopaedic surgeon who happens to perform damage control surgery as part of wider responsibilities.

15. How many hours a week is a Full Time Equivalent (FTE) in your hospital:
For doctors …………. hours
For nurses ……………hours

FTE = Full time equivalent. This will provide the basis for calculation of staffing resources in subsequent questions. In these subsequent questions '1 FTE' may be constituted by one person who works on a fulltime basis, but can also refer to two persons who work half-time.<br><br>We are aware that many physicians undertake a great deal of overtime work (weekend, night shifts) and often work more than is stated in their contract. For this question we are not interested in how many hours they actually work, but in how many hours they work according to their contract.

16. How many neurosurgeons (in FTE) work at your hospital?
……………….FTE neurosurgeons

……………… FTE neurosurgery trainees in residency training
…………….. FTE neurosurgery trainees not in residency training

The amount of FTEs do not have to be a whole number. If the amount of FTE is, for example, 3.3, please write down '3.3' here and not '3'!<br>If there are persons with out of hours work that is contracted and paid for, you can count them as > 1 FTE. For example, if there is a physician that is paid for 60 hours a week and 48 hours a week is considered as a FTE for a doctor in your hospital, you can count this physician as 60/48 = 1.25 FTE<br><br>The term 'trainee not in residency training' refers to a clinician working in your hospital who is not qualified as a specialist, but is also not part of a formal training scheme towards becoming a specialist (neurosurgeon in this case).

17. How many neurologists (in FTE) work at your hospital?
……………….FTE neurologists
……………….FTE neurologist trainees in residency training
……………….FTE neurologist trainees not in residency training

The amount of FTEs do not have to be a whole number. If the amount of FTE is, for example, 3.3, please write down '3.3' here and not '3'!<br>If there are persons with out of hours work that is contracted and paid for, you can count them as > 1 FTE. For example, if there is a physician that is paid for 60 hours a week and 48 hours a week is considered as a FTE for a doctor in your hospital, you can count this physician as 60/48 = 1.25 FTE<br><br>The term 'trainee not in residency training' refers to a clinician working in your hospital who is not qualified as a specialist, but is also not part of a formal training scheme towards becoming a specialist (neurologist in this case)

18. How many trauma surgeons (in FTE) work at your hospital?
……………….FTE trauma surgeons
……………….FTE trauma surgeon trainees in residency training
……………….FTE trauma surgeon trainees not in residency training

The amount of FTEs do not have to be a whole number. If the amount of FTE is, for example, 3.3, please write down '3.3' here and not '3'!<br>If there are persons with out of hours work that is contracted and paid for, you can count them as > 1 FTE. For example, if there is a physician that is paid for 60 hours a week and 48 hours a week is considered as a FTE for a doctor in your hospital, you can count this physician as 60/48 = 1.25 FTE<br><br>The term 'trainee not in residency training' refers to a clinician working in your hospital who is not qualified as a specialist, but is also not part of a formal training scheme towards becoming a specialist (trauma surgeon in this case)<br><br>In this response, the term 'trauma surgeon' refers to an individual who specializes in trauma surgery, not a general surgeon or orthopaedic surgeon who happens to perform damage control surgery as part of wider responsibilities

19. How many intensivists (in FTE) work at the Intensive Care Unit (ICU) in your hospital? (all ICUs together if you have multiple):
……………….FTE intensivists
……………….FTE intensivist trainees in residency training
……………….FTE intensivist trainees not in residency training

The amount of FTEs do not have to be a whole number. If the amount of FTE is, for example, 3.3, please write down '3.3' here and not '3'!<br>If there are persons with out of hours work that is contracted and paid for, you can count them as > 1 FTE. For example, if there is a physician that is paid for 60 hours a week and 48 hours a week is considered as a FTE for a doctor in your hospital, you can count this physician as 60/48 = 1.25 FTE<br>The term 'trainee not in residency training' refers to a clinician working in your hospital who is not qualified as a specialist, but is also not part of a formal training scheme towards becoming a specialist (intensivist in this case)

20. How many Emergency Department (ED) physicians (in FTE) work at your ED?
……………………FTE ED physicians
……………………FTE trainees in residency training
……………………FTE trainees not in residency training

The amount of FTEs do not have to be a whole number. If the amount of FTE is, for example, 3.3, please write down '3.3' here and not '3'!
If there are persons with out of hours work that is contracted and paid for, you can count them as > 1 FTE. For example, if there is a physician that is paid for 60 hours a week and 48 hours a week is considered as a FTE for a doctor in your hospital, you can count this physician as 60/48 = 1.25 FTE

The term 'trainee not in residency training' refers to a clinician working in your hospital who is not qualified as a specialist, but is also not part of a formal training scheme towards becoming a specialist (ED physician in this case)

Payments

21. What is the system of funding that supports your hospital?
  - o Government / Public
  - o Private (includes both for-profit and non-profit institutions)

For the purposes of this question:
A government or public hospital is one that is owned by the government and receives government funding.
A private hospital is owned by a commercial organization or a non-profit organization and privately funded through payment for medical services by patients themselves, by health care insurers, or by the government through national health insurance schemes.

22. How are doctors who treat Traumatic Brain Injury (TBI) patients paid in your hospital?
  - o Doctors have a fixed monthly salary
  - o Fee for services (Doctors are paid per activity; for example, per operation, per investigation, per hospital day, etc.)
  - o Doctors are paid per patient (they receive a fixed amount of money per diagnosis)
  - o Doctors have a fixed monthly salary and are paid an extra fee for services
  - o Other, please specify____

This question seeks information on all doctors that treat TBI patients in your hospital, such as ED physicians, trauma surgeons, neurologists, neurosurgeons and intensivists.

If there is a difference between doctors, please select other and explain what doctors receive what form of payment. For example: "Intensivists and trauma surgeons have a fixed monthly salary, neurosurgeons are paid per operation"

A standard salary supplement for out of hours work, either resident in hospital or on call, should be interpreted as part of a fixed salary rather than extra fee for service

23. Does the salary of the doctor depend on a patient's insurance coverage?
  - o No
  - o Yes
  - o NA. all inhabitants in our country are insured

For example: If a patient is uninsured or insured by a particular insurance company, the doctor may receive less payment for the same operation in comparisons to patients who are insured or insured by another insurance company

24. Do doctors (or the department) receive additional payments for treatment of "private" patients and/or for patients who opt for extra facilities such as a single room?
  - o No
  - o Yes

"additional payments" do not need to be paid directly to the doctor as part of his/her personal salary, but may be paid into a departmental or personal fund which could include, for example, reimbursement of professional costs, including attendance at conferences, travel, dinner expenses etc.

Equipment

25. Do you have an electronic patient record in your hospital as a whole (not just confined to the ICU)?
- No
- Yes

“Electronic patient record” refers to a system that stores all patient information (for example laboratory values, CT scans, observatory notes, letters to the GP) electronically and not in a paper format.

26. Do you have an electronic data system in your Intensive Care Unit (ICU)?
- No
- Yes

Please notice: this question is only related to your ICU and not to your hospital as a whole (see previous question)

27. Is a Computed Tomography (CT) technician/radiographer available 24/7 to perform a CT scan?
- No.
- Yes. There is 24/7 in-house availability of a resident CT technician/radiographer to perform CT scans
- Yes. CT technician/radiographers are on call and would arrive within 30 minutes to perform CT scans
- Yes. CT technician/radiographers are on call, but would only arrive after 30 minutes to perform CT scans

28. Is a Magnetic Resonance Imaging (MRI) technician/radiographer available 24/7 to perform a MRI scan?
- No.
- Yes. There is 24/7 in-house availability of a resident MRI technician/radiographer to perform MRI scans
- Yes. MRI technician/radiographers are on call and would arrive within 30 minutes to perform MRI scans
- Yes. MRI technician/radiographers are on call, but would only arrive after 30 minutes to perform MRI scans

29. Is S100B routinely determined as a prognostic biomarker for neurological deterioration?
- No
- Yes

Hospital Costs

30. What are the average daily reimbursement costs for a severe Traumatic Brain Injury (TBI) patient in your hospital?

…………………euro

Reimbursement costs are the total costs that can be claimed from the insurance provider/the patient/public or government funds<br><br>If your country uses another monetary unit than the Euro, please calculate as Euros using a current exchange rate!

31. What are the average reimbursement costs of an Emergency Department visit?

………… euro

Reimbursement costs are the total costs that can be claimed from the insurance provider/the patient/public or government funds

If your country uses another monetary unit than the Euro, please calculate as Euros using a current exchange rate!

32. What are the average reimbursement costs of one day hospitalization at the ward?

……………euro

Reimbursement costs are the total costs that can be claimed from the insurance provider/the patient/public or government funds

If your country uses another monetary unit than the Euro, please calculate as Euros using a current exchange rate!

33. What are the average reimbursement costs one day hospitalization at the Intensive Care Unit (ICU)? (excluding the coronary care unit)?

……………..euro

A coronary care unit refers to a unit or some ICU beds that are specialized in patients with acute heart or coronary diseases. Reimbursement costs are the total costs that can be claimed from the insurance provider/the patient/public or government funds.

If your country uses another monetary unit than the Euro, please calculate as Euros using a current exchange rate!

34. What are the reimbursement costs for a routine clinical (not research) CT scan of the head for an ambulant patient in your hospital?

……………………..euro

Reimbursement costs are the total costs that can be claimed from the insurance provider/the patient/public or government funds

If your country uses another monetary unit than the Euro, please calculate as Euros using a current exchange rate!

35. What are the reimbursement costs for a routine clinical (not research) MRI scan of the head for an ambulant patient in your hospital?

……………………euro

Reimbursement costs are the total costs that can be claimed from the insurance provider/the patient/public or government funds

If your country uses another monetary unit than the Euro, please calculate as Euros using a current exchange rate!

36. What are the reimbursement costs for an ICP monitoring catheter in your hospital?

…………… euro for a parenchymal ICP monitoring catheter (device only)

…………… euro for ventricular catheter (device only)

Reimbursement costs are the total costs that can be claimed from the insurance provider/the patient/public or government funds

If your hospital never use respectively a parenchymal or ventricular catheter, you can type NA in the boxes.

If your country uses another monetary unit than the Euro, please calculate as Euros using a current exchange rate!

37. What is the average fulltime (1FTE) gross yearly base salary for a:

- Newly qualified nurse ………………Euro
- Senior nurse in charge of a ward during shifts ……………Euro

- Senior nurse in charge of an intensive care during shifts …………..Euro
- First year neurosurgery trainee in residency program: …………………………. Euro
- Final year neurosurgery trainee in residency program:……………………………… Euro
- First year intensive care trainee in residency program…………………..Euro
- Final year intensive care unit trainee in residency program…………………..Euro
- First year emergency medicine trainee in residency program…………………..Euro
- Final year emergency medicine trainee in residency program…………………..Euro

Please provide us the base salary only (without compensation fees, bonuses etc).<br>With a senior nurse in charge of a ward/ICU during shifts we refer to a senior nurse who is in charge of respectively the ward and the ICU during a part of the day (morning shift, night shift), but not in managerial charge of the ward/ICU as an organization.<br>A first year trainee in residency program is someone who finished medical school and just started with the training towards becoming a specialist. There may be differences among different specialisms (trauma surgeon, neurologist), so please give us an estimate here.<br>The last year trainee in residency program is someone who is almost ready with the residency program and who will become a specialist (eg neurosurgeon, trauma surgeon) in the next year.<br>If your country uses another monetary unit than the Euro, please calculate as Euros using a current exchange rate!

38. Where did you find this information?
…………………………………………………………..

Name the source: for example annual report, registry

Acute Trauma care

39. Is there an in-hospital multidisciplinary team which will be alerted if a serious trauma victim comes in?

- ☐ No
- ☐ Yes

Some hospitals have a multidisciplinary team that is called when a serious trauma victim is expected. The function is early triage and treatment

40. Do you have a helicopter platform for helicopter trauma medical services?

- ☐ No
- ☐ Yes

40b. . How often is the helicopter platform approximately used per month?
………….. times

Teleconsulting and transfer

41. Which specialist usually makes the decision about transfer of Traumatic Brain Injury (TBI) patients to your hospital?

- ☐ Intensivist
- ☐ Neurosurgeon
- ☐ Trauma surgeon
- ☐ General or orthopaedic surgeon
- ☐ Neurologist
- ☐ Other, please specify……………………………………………..

This is the specialist who is often contacted by telephone or teleconsult and who decides that a TBI patient in another hospital will be transferred to your hospital<br><br>In this response, the term ‘trauma surgeon’ refers to an individual who specializes in trauma surgery, not a general surgeon or orthopaedic surgeon who happens to perform damage control surgery as part of wider responsibilities.

Information about the completer of the questionnaire

Other than the CENTER-TBI investigator, which of the following individuals was involved in completion of this questionnaire?

Tick all that apply

- ☐ Neurologist
- ☐ Neurosurgeon
- ☐ Trauma Surgeon
- ☐ Emergency Department (ED) physician
- ☐ Administrative staff member / data manager / financial department
- ☐ Other, please specify……………..
- ☐ NA. The questionnaire was completed soley by the CENTER TBI local investigator

Medical Ethics

1.Does your institute have a department of medical ethics?

- o No
- o Yes
- o Unknown

With a department of medical ethics we mean an academic department of ethics and philosophy or medicine, and not an office for institutional reviews of research documents. The department of medical ethics stands for research and education in medical ethics and philosophy of medicine. Usually the ethicists and faculty members in the department collaborate with clinicians on topics in different research areas in biomedical ethics

2.Is a medical ethicist available in your institution for consultation on patient care?

- o No
- o No, but a cleric (for example a priest, spiritual care) with ethical knowledge
- o No, but a physician with ethical expertise
- o Yes, a certified medical ethicist (involved in ethics consultation, research and education)
- o Yes, but only for deliberation on end-of-life issues
- o Other, please specify………………………………………………

3.Does your country have a *central* ethics committee for evaluation of research proposals?

- o No
- o Yes
- o Unknown

3b.If yes, what type of studies should be submitted to this committee?
Tick all that apply

- ☐ Phase I clinical trials (healthy volunteers)
- ☐ Phase II clinical trials testing experimental therapeutic interventions
- ☐ Phase III clinical trials testing therapeutic interventions
- ☐ Observational studies in mentally incapacitated patients
- ☐ Observational studies in emergency situations
- ☐ Intervention studies in mentally incapacitated patients
- ☐ Therapeutic studies (e.g. involving drugs) in children
- ☐ Non-therapeutic studies in children
- ☐ Studies involving genetics

- ☐ Studies involving vaccines

4.Can an Institutional Review Board (IRB) approval which is obtained in one centre be used to facilitate approval of submissions in other participating centres in your country?

- No
- Yes

An IRB (Institutional review board) is a multidisciplinary committee that evaluates research protocols and provides a binding judgment on whether the project is permissible on ethical grounds

4b.If yes,

- Yes, approval will follow without detailed evaluation
- Yes, but additional detailed evaluation is required
- Unknown

5.Does your IRB make any distinction in decision making for studies on acutely mentally incapacitated patients (e.g. TBI, stroke, cardiac arrest) or chronically mentally incapacitated patients (e.g. serious psychiatric disorders, dementia)?

- No
- Yes
- Unknown

6.Is there a consistent IRB policy for evaluations and approvals of studies in your country or are there to your knowledge variations between regions/cities/centres?

- Consistent policy
- Mainly consistent but there may be some variations
- Substantial variations exist between different states or cities
- Large variations exist strongly dependant on local IRB's

7.How many IRB applications have you / your department submitted over the past five years?

- Zero
- 1-2
- 3-5
- > 5
- Unknown

8.How many IRB applications have you / your department submitted over the past five years for studies of patients with acute cerebral disorders (involving mentally incapacitated patients in emergency situations)?

- Zero
- 1-2
- 3-5
- > 5
- Unknown

8b.Did you obtain approval in all submissions?

- No
- Yes
- Unknown

9.What is the average turnover time in your centre for IRB evaluations?

- 1 month
- 2 months

- 3 months
- 4 months
- 5 months
- 6 months
- > 6 months

10.Is proxy consent by a close relative considered valid by your IRB in acutely mentally incapacitated patients (e.g. TBI)?

- No
- Yes
- Unknown

11.Is proxy consent by a close relative considered valid by your IRB in chronic mentally incapacitated patients (e.g. psychiatric patients)?

- No
- Yes
- Unknown

12.Is clinical research involving mentally incapacitated patients in emergency situations regulated by law in your country?

- Yes, it is incorporated in national law
- No, it is not regulated by law
- No, it is not regulated by law, but we follow European Union regulations
- No, but we follow Good Clinical Practice or Declaration of Helsinki guidelines
- No, it is not regulated by law, but IRBs have the authority to approve
- Other, please specify……………………………………………
- I don't know

13.Can consent by waived or deferred in emergency clinical research in your **country**?

- Yes, this is regulated by law
- Yes, after prior approval of an IRB
- No
- I don't know

14.Is proxy consent by family members considered valid for mentally incapacitated patients in your **country**?

- No
- Yes
- Unknown

15.Is consent by an independent physician for participation of a mentally incapacitated patient considered valid in your **country**?

- No
- Yes
- Unknown

16.Do you have experience with obtaining IRB approval for emergency research involving mentally incapacitated patients?

- No
- Yes

16b.If yes, have the IRBs in your **country** made objections to emergency research involving mentally incapacitated patients without prior consent of patient or legal representatives?

- No
- Sometimes
- Yes

16c.If the IRB objected to emergency research involving mentally incapacitated patients without prior patient- or proxy consent, on which grounds or argumentation?

- ☐ It is against the law in this country
- ☐ Only a judge or other official juridical organ can approve such kind of research
- ☐ Only a patient him/herself can consent for inclusion in a study
- ☐ Prior written consent of a legal representative is always necessary before inclusion in a study
- ☐ Other, please specify ……………………………………

17.Do you think that emergency research involving mentally incapacitated patients without prior patient- or proxy consent is ethically feasible?

- No
- Yes

The responses to this question should represent, as best as practicable, a general consensus, rather than your personal opinion

17b.If yes, on which grounds?

- ☐ The emergency nature of the situation and possible harm to the patient
- ☐ Obtaining consent from relatives in distress is not valid
- ☐ Valuable possible therapeutic time is lost with the obligation to obtain consent

Other, please specify……………..

Provider Profiling Questionnaire

Questionnaire 7: treatment at the Intensive Care Unit (ICU)

This questionnaire can be completed by a(n) (neuro)intensivist in collaboration with a neurosurgeon

For the completion of this questionnaire, we advise you to ask help from a data manager, administrative staff member and/or someone from the financial department in your hospital, since we ask for hospital data in this questionnaire. It is very important that this information is accurate, and searched for in annual reports, registries and other data sources rather than estimated.

This questionnaire also includes questions about the general policy in your hospital. The responses to these questions should represent, as best as practicable, a general consensus on treatment at your centre, rather than individual management preferences. Consequently, you should provide responses that describe not what you would do personally, but how the majority of patients would generally be treated in your centre.

There are no 'right' or 'wrong' answers so please give us a realistic and honest view of how the care in your hospital is organized. Your answers will only be used to answer the scientific questions in CENTER TBI and no information in any form will be reported on individual centre level. Some of the questions may seem similar, but please answer all questions.

If you have any questions or problem, please contact:
Maryse Cnossen, PhD student (m.c.cnossen@erasmusmc.nl)

Information about the completer of the questionnaire

Other than the CENTER-TBI investigator, which of the following individuals was involved in completion of this questionnaire?

Select all that apply

- ☐ Neurologist
- ☐ Neurosurgeon
- ☐ Trauma Surgeon
- ☐ ED physician
- ☐ Administrative staff member / data manager / financial department
- ☐ Other, please specify…………….
- ☐ NA. The questionnaire is solely completed by the CENTER TBI local investigator

The Local investigator is the senior clinician(s) at your hospital involved in supervision of CENTER TBI

General patient statistics

What is the number of patients treated in your Intensive Care Unit (ICU) annually?

1. 2012: …………………………………….
2. 2013: …………………………………….

What is the number of Traumatic Brain Injury (TBI) patients treated in your Intensive Care Unit (ICU) annually?

3. 2012: …………………………………….
4. 2013: …………………………………….

5.In how many patients with Traumatic Brain Injury (TBI) did you perform ICP monitoring last year (2013)?

…………………………………………….

6.Where did you find this information?

………………………………………………………..

Name the source: for example annual report, registry

Indications for admission to your Intensive Care Unit (ICU)

7. Which Traumatic Brain Injury (TBI) patients are admitted to your Intensive Care Unit (ICU)?

Please provide us the general clinical practice at your centre. This does not have to be the same as stated in the guidelines you use

This question is about indications for ICU admission in patients with TBI.<br><br>Select CONSIDERED NOT IMPORTANT IN DECISION MAKING in factors that are never a reason for ICU admission.<br><br>Select ONLY IN THE PRESENCE OF OTHER RISK FACTORS if the factor is never solely a reason ICU admission, but it might be a reason in combination with one or more other risk factors. For example: a hospital may only admit patients with a GCS ≤ 8 if they have an abnormal head CT as well. Respondents from such a hospital should tick ‘only in the presence of other risk factors’ after GCS ≤ 8

Select GENERAL POLICY when the criteria are, in general, a reason for ICU admission in your hospital. When you select GENERAL POLICY this must represent a general consensus among colleagues, rather than individual preference.
Where you are in doubt whether this is the appropriate response to the question, we would recommend, for example, either a verbal discussion or an email exchange with colleagues to check consensus.

	Considered not important in decision making	Only in the presence of other risk factors	General Policy
GCS ≤ 8	☐	☐	☐
GCS 9-12 with CT abnormalities	☐	☐	☐
GCS 9-12 without CT abnormalities	☐	☐	☐
GCS 13-15 with CT abnormalities but no large structural lesion	☐	☐	☐
GCS 13-15 with small EDH or ASDH	☐	☐	☐
GCS 13-15 with contusional brain damage	☐	☐	☐
anti-coagulant therapy in patients with GCS 13-15	☐	☐	☐
After intracranial surgery	☐	☐	☐
Mild TBI with concomitant extracranial injuries that in isolation, would not necessitate ICU admission	☐	☐	☐

Use of protocols / guidelines

8. With reference to guidelines for Intensive Care Unit (ICU) management of Traumatic Brain Injury (TBI), does your ICU:

- Not have specific guidelines for management
- Follow the Brain Trauma Foundation Guidelines
- Follow National Guidelines (Please specify: ……………………………………………)
- Have institutional guidelines which are broadly based on BTF and/or National Guidelines
- Have separate guidelines which you have developed independently

(if #4 or #5 above, please upload a copy of your guidelines)

9. If guidelines for Traumatic Brain Injury (TBI) management exist in your Intensive Care Unit (ICU), how are they implemented?

Select all that apply

- ☐ No formal implementation of guidelines
- ☐ Verbal direction from clinical managers/ clinical directors/senior doctors
- ☐ Written protocols and algorithms
- ☐ Training organised by an external organisation
- ☐ Training organised by your own hospital / ICU
- ☐ E-learning
- ☐ flowchart / algorithms / protocols in the patient data management system of your ICU
- ☐ Periodic feedback on adherence to the guideline
- ☐ Structural attention for protocol adherence during clinical rounds
- ☐ Other (please specify) ……………………………………………………………………………….

10. Is there a group or individuals who oversee guideline development and maintenance?

- ☐ Neither
- ☐ Individual
- ☐ Group
  - o Single discipline: ICU/neurosurgery/neurology (please circle correct response)
  - o Multidisciplinary

11. Have there been audits to check guideline adherence in your Intensive Care Unit (ICU)?

- o Not in last five years
- o Once in the last five years
- o Annually
- o Several times per year

An audit is a process in which the hospital/ICU assesses how well guidelines are followed.

12. Please estimate Traumatic Brain Injury (TBI) guideline adherence for:

	Never (0-10%)	Rarely (10-30%)	Sometimes (30-70%)	Frequently (70-90%)	Always (90-100%)
For medical management	☐	☐	☐	☐	☐
For surgery	☐	☐	☐	☐	☐

The responses to this question should represent, as best as practicable, a general consensus, rather than individual thoughts.

13. What are reasons for non-adherence to guidelines in patients with Traumatic Brain Injury (TBI) ?

	Never (0-10%)	Rarely (10-30%)	Sometimes (30-70%)	Frequently (70-90%)	Always (90-100%)
Lack of knowledge among clinicians	☐	☐	☐	☐	☐

Every patient is unique and should be managed by clinical judgment	☐	☐	☐	☐	☐
Inadequate time to consult guidelines for urgent decisions	☐	☐	☐	☐	☐
Guidelines on TBI do not apply due to extracranial trauma or comorbidity	☐	☐	☐	☐	☐
Inadequate resources to apply guidelines (ICU beds, personnel, equipment)	☐	☐	☐	☐	☐
Other, please specify………………	☐	☐	☐	☐	☐

The responses to this question should represent, as best as practicable, a general consensus, rather than individual thoughts.

Monitoring

Routinely repeated CT scans

In this context "routine repeat CT scan" refers to CT scans that are scheduled at the beginning of the ICU admission, and undertaken at predetermined time points regardless of clinical situation.

14. Do you use routinely repetitive CT scanning in patients with Traumatic Brain Injury (TBI)?

- No
- Yes

14b. If yes: What are indications for routinely repetitive CT scanning in TBI patients admitted to the ICU? *Please provide us the general clinical practice at your centre. This does not have to be the same as stated in the guidelines you use*

This question is about indications for routinely repetitive CT scanning in TBI patients.

Select CONSIDERED NOT IMPORTANT IN DECISION MAKING in factors that are never a reason for routinely repetitive CT scanning.

Select ONLY IN THE PRESENCE OF OTHER RISK FACTORS if the factor is never solely a reason for routinely repetitive CT scanning, but it might be a reason in combination with one or more other risk factors. For example: a hospital may schedule routinely repetitive CT scanning in patients with severe TBI on coagulant medication. Respondents from such a hospital should tick 'only in the presence of other risk factors' after GCS ≤ 8 and 'patients on anticoagulants/antiplatelet medication'.

Select GENERAL POLICY when the criteria are, in general, a reason for routinely repetitive CT scanning in your hospital. When you select GENERAL POLICY this must represent a general consensus among colleagues, rather than individual preference.

Where you are in doubt whether this is the appropriate response to the question, we would recommend, for example, either a verbal discussion or an email exchange with colleagues to check consensus.

	Considered not important in decision making	Only in the presence of other risk factors	General Policy
Routinely in all TBI cases on the ICU (e.g. 24 or 48 hours post ICU admission)	☐	☐	☐
In all patients with intracranial lesions	☐	☐	☐
In all patients with admission GCS ≤ 8	☐	☐	☐
Patients on anticoagulants/antiplatelet medication	☐	☐	☐
Following intracranial surgery	☐	☐	☐
A history of substance abuse	☐	☐	☐

14c. If you use routinely repetitive CT scanning in TBI patients. What is the typical time scheme?

Scan 1: …….hours after the first CT scan
Scan 2: …….hours after the first CT scan
Scan 3: …….hours after the first CT scan
Scan 4: …….hours after the first CT scan
Scan 5: …….hours after the first CT scan
Scan 6: …….hours after the first CT scan

ICP monitoring

15. What are indications for ICP monitoring in your hospital?
Please provide us the general clinical practice at your centre. This does not have to be the same as stated in the guidelines you use

This question is about indications for ICP monitoring in your centre.

Select CONSIDERED NOT IMPORTANT IN DECISION MAKING in factors that are never a reason for ICP monitoring.

Select ONLY IN THE PRESENCE OF OTHER RISK FACTORS if the factor is never solely a reason for ICP monitoring, but it might be a reason in combination with one or more other risk factors. For example: a hospital may perform ICP monitoring in patients with a GCS ≤ 8 without CT abnormalities if there are other risk factors present. Respondents from such a hospital should tick 'only in the presence of other risk factors' after GCS ≤ 8.

Select GENERAL POLICY when the criteria are, in general, a reason for ICP monitoring in your hospital. When you select GENERAL POLICY this must represent a general consensus among colleagues, rather than individual preference.

Where you are in doubt whether this is the appropriate response to the question, we would recommend, for example, either a verbal discussion or an email exchange with colleagues to check consensus.

	Considered not important in decision making	Only in the presence of other risk factors	General Policy
GCS ≤ 8 and CT abnormalities	☐	☐	☐
GCS ≤ 8 without CT abnormalities	☐	☐	☐
GCS 9-12 with contusion	☐	☐	☐
Inability to assess a patient with CT abnormalities clinically (e.g. sedation, surgery etc.)	☐	☐	☐
Intraventricular haemorrhage	☐	☐	☐
Other, please specify…………………………	☐	☐	☐

16. What are reasons for NOT monitoring ICP at your Intensive Care Unit (ICU)?

	Never (0-10%)	Rarely (10-30%)	Sometimes (30-70%)	Frequently (70-90%)	Always (90-100%)
Glasgow Coma Scale (GCS) > 8	☐	☐	☐	☐	☐
No radiological signs of raised ICP	☐	☐	☐	☐	☐
Risk of raised ICP considered low	☐	☐	☐	☐	☐

Patient considered unsalvageable	☐	☐	☐	☐	☐
Coagulopathy (non-drug related)	☐	☐	☐	☐	☐
Use of anticoagulants or platelet aggregation inhibitors	☐	☐	☐	☐	☐
No device available	☐	☐	☐	☐	☐
Not local policy to monitor ICP	☐	☐	☐	☐	☐
We adhere to a protocol in which treatment is based on imaging and clinical examination	☐	☐	☐	☐	☐
Too costly	☐	☐	☐	☐	☐
Other, please specify……	☐	☐	☐	☐	☐

> The responses to this question should represent, as best as practicable, a general consensus on treatment at your centre, rather than individual management preferences or opinions.

17. Is there structural variation between (neuro)surgeons within your hospital with regard to the decision to place an ICP sensor?

- o No
- o Yes

> Structural variation refers to a situation in which one or more of the neurosurgeons are generally more likely to place an ICP sensor than others.

18. When a patient with polytrauma and minor intracranial pathology (which would not otherwise indicate ICP monitoring) requires <u>extracranial surgery</u> which is not life-saving, in the acute phase after trauma, do you:

Select all that apply

- ☐ Place an ICP monitor and allow surgery to proceed
- ☐ Repeat a CT scan before/after surgery
- ☐ Undertake a sedation hold before and/or after surgery
- ☐ Postpone surgery if at all possible
- ☐ Other, please specify…..

> The responses to this question should represent, as best as practicable, a general consensus on treatment at your centre, rather than individual management preferences.

19. In polytrauma patients with a Glasgow Coma Scale (GCS) >8 and small but not severe initial CT abnormalities, who require <u>mechanical ventilation</u> for a number of days because of extracranial injuries, we apply ICP monitoring:

- o Never
- o Only sometimes
- o Often
- o Always

> The responses to this question should represent, as best as practicable, a general consensus on treatment at your centre, rather than individual management preferences.

20. What kind of ICP sensors are used in your hospital?

- o Parenchymal monitors without optional ventricular drainage
- o Ventricular catheters
- o Both
- o Not applicable since ICP sensors are not used

20b. In case you answered 'both' in the previous question:
If you use parenchymal and ventricular catheters in your hospital, when would you use ventricular/ventricular+ sensor monitors (instead of parenchymal monitoring)?

please rank the top 3 reasons:
1: …………..
2: …………
3: …………..

- ☐ Routine in our department
- ☐ Not routine, but enlarged ventricles
- ☐ External CSF drainage
- ☐ No parenchymal device available
- ☐ Low cost
- ☐ Other, please specify……………………………………

20c. If you use parenchymal and ventricular catheters in your hospital, when would you use parenchymal monitors (instead of ventricular/ventricular+sensor)?

1: …………..
2: …………
3: …………..

- ☐ Routine in our department
- ☐ Not routine, but small ventricles
- ☐ Mainly motivated by time of day
- ☐ No OR available for placement ventricular catheter
- ☐ Failed implantation ventricular cathether
- ☐ Other, please specify………………………………..

20d. When deciding to monitor ICP we routinely use additional ventricular CSF drainage:

- o No, never or seldom
- o As second tier therapy to control ICP
- o Only if ventricles are enlarged
- o Yes, always initially

20e. If you use a ventricular drain:

During the use of the ventricular drain, is the drain open or closed during the most of the time?

- ☐ Ventricular drain is open to drain CSF
  - o If this answer option is selected: At what level? ……………………………………………
- ☐ Ventricular drain is closed mostly and opened intermittently
  - o If this answer option is selected: At what pressure is this opened? And for how long?

☐ Other, please specify……………………………..

Intensive Care Unit (ICU) practice around ICP monitoring

	Never (0-10%)	Rarely (10-30%)	Sometimes (30-70%)	Frequently (70-90%)	Always (90-100%)	N/A, we do not have this technique
21. Are prophylactic antibiotics given prior to ICP monitor insertion?	Ventricular catheter:					
	○	○	○	○	○	○
	Parenchymal sensor:					
	○	○	○	○	○	○
22. Are prophylactic antibiotics continued after ICP monitoring insertion?	Ventricular catheter:					
	○	○	○	○	○	○
	Parenchymal sensor					
	○	○	○	○	○	○
23. Is a coagulation panel assessed prior to insertion of an ICP monitoring device?	Ventricular catheter:					
	○	○	○	○	○	○
	Parenchymal sensor					
	○	○	○	○	○	○

24. What is considered a minimum platelet count for insertion of a ventricular catheter in your Intensive Care Unit (ICU)?

- ○ >150K
- ○ >100K
- ○ > 80 K
- ○ >50K
- ○ Variable, depends on surgeon
- ○ No minimum
- ○ Other, please specify …………………………………..

25. What is consider the minimum INR for safe placement of a ventricular catheter in your Intensive Care Unit (ICU)?

- <1.4
- <1.3
- <1.2
- Variable, depending on surgeon
- No minimum
- Other, please specify………………………………………………………………..

26. Who inserts the catheter/probes for ICP monitoring?
Please provide us the general clinical practice at your centre. This does not have to be the same as stated in the guidelines you use

This question is about indications for ICP monitoring in your centre.

Select NEVER if the specialism has never inserted ICP monitors in TBI patients.

Select RARELY / EXCEPTIONAL if the specialism CAN insert ICP monitors and does this during exceptional circumstances (e.g. during the night, crisis, overcrowding).

Select GENERAL POLICY when the specialism usually inserts ICP monitors.

Where you are in doubt whether this is the appropriate response to the question, we would recommend, for example, either a verbal discussion or an email exchange with colleagues to check consensus.

	Never	Rarely / Exceptional	General Policy
Neurosurgeon	☐	☐	☐
Neurosurgical resident	☐	☐	☐
Intensivist	☐	☐	☐
Intensivist resident	☐	☐	☐
Neurointensivist	☐	☐	☐
Non-neurosurgical surgeon	☐	☐	☐
Physician assistant / nurse practitioner	☐	☐	☐
Other, please specify….	☐	☐	☐

27. At what level does your Intensive Care Unit (ICU) zero the ICP catheter?
- Foramen of Monro
- Same level as arterial blood pressure
- Other, please specify……………………………..

28. At what level does your Intensive Care Unit (ICU) zero the transducer for arterial blood pressure (for calculation of CPP)?
- Right atrium
- Level of arterial catheter
- Foramen of Monroe
- Other, please specify………………..

29. How is the alignment of the transducer to the chosen reference level checked?
- Not formally checked
- By eye
- Spirit level

- Laser indication
- Other (please specify)………………..

29b. If the level is formally assessed, is this done:

- At admission
- Once each day
- Once per nursing shift
- More frequently

CPP monitoring

30. Please list the target Cerebral Perfusion Pressure utilized at your facility:
Select all that apply

- ☐ > 50 mmHg
- ☐ > 60 mmHg
- ☐ > 70 mmHg
- ☐ Individualized

31. For treating CPP, which types of IV fluids are used to augment intravascular volume?
Select all that apply

- ☐ Crystalloids
- ☐ Colloids – starches
- ☐ Colloids - albumin
- ☐ Other combinations

32. Which vasoactive drugs are used to support CPP in patients with Traumatic Brain Injury (TBI)?
Select all that apply

- ☐ Vasopressors
- ☐ Inotropes
- ☐ Other, please specify………

33. What monitoring devices are used to titrate vasoactive drugs?
Select all that apply

- ☐ MAP targets only
- ☐ Central venous pressure
- ☐ PICO
- ☐ Lidco
- ☐ Oesophageal Doppler monitor
- ☐ Pulmonary artery catheter
- ☐ Others, please specify ……

Advanced monitoring

34. Which of the following additional techniques are utilized at your Intensive Care Unit (ICU) for neuromonitoring?
Select all that apply

	Never (0-10%)	Rarely (10-30%)	Sometimes (30-70%)	Frequently (70-90%)	Always (90-100%)
Cerebral	☐	☐	☐	☐	☐

microdialysis					
Transcranial Doppler	☐	☐	☐	☐	☐
CBF probes	☐	☐	☐	☐	☐
Jugular Venous saturation monitors	☐	☐	☐	☐	☐
Near infrared monitors	☐	☐	☐	☐	☐
Brain tissue oxygenation	☐	☐	☐	☐	☐
Other, please specify………….	☐	☐	☐	☐	☐

Treatment of severe Traumatic Brain Injury (TBI) patients at the Intensive Care Unit (ICU)

35. At your Intensive Care Unit (ICU), the threshold for medical management of elevated ICP is:
- > 15 mmHg
- >20 mmHg
- >25 mmHg
- Other, please specify……………..

36. At your Intensive Care Unit (ICU), the threshold for performing a decompressive craniectomy in patients with diffuse injury is:
- > 15 mmHg
- >20 mmHg
- >25 mmHg
- > 30 mm Hg
- NA. Decompressive craniectomy is (almost) never performed in our hospital

Sedation

37. Please rate the utilization of the following sedatives, neuromuscular blockers, and analgesics <u>as first line therapies</u> for ICU management of Traumatic Brain Injury (TBI) patients with increased ICP:

	Never (0-10%)	Rarely (10-30%)	Sometimes (30-70%)	Frequently (70-90%)	Always (90-100%)
Fentanyl	☐	☐	☐	☐	☐
Midazolam	☐	☐	☐	☐	☐
Morphine (or other opioids)	☐	☐	☐	☐	☐
Propofol	☐	☐	☐	☐	☐
Neuromuscular blocking agents	☐	☐	☐	☐	☐
Alfa 2 agonist (clonidine or dexmedetomidine)	☐	☐	☐	☐	☐
Barbiturates	☐	☐	☐	☐	☐

Other, please specify…..	☐	☐	☐	☐	☐

Hyperosmolar therapy (mannitol and/or hypertonic saline
Please provide us the general clinical practice at your centre. This does not have to be the same as stated in the guidelines you use

> This question is about the use of hyperosmotic therapy in patients with elevated ICP.
>
> Select NEVER in agents that are never used in your centre.
>
> Select RARELY / EXCEPTIONAL in agents that can be used but are no general policy.
>
> Select GENERAL POLICY when the agent is, in general, used to treat elevated ICP in your centre.
> When you select GENERAL POLICY this must represent a general consensus among colleagues, rather than individual preference.
> Where you are in doubt whether this is the appropriate response to the question, we would recommend, for example, either a verbal discussion or an email exchange with colleagues to check consensus.

	Never	Rarely / Exceptional	General Policy
38. Is mannitol utilized to treat patients with increased ICP in your ICU ?	☐	☐	☐
39. Is hypertonic saline utilized to treat patients with increased ICP in your ICU?	☐	☐	☐
40. Is hypertonic saline administered in conjunction with mannitol?	☐	☐	☐

Mannitol and Hypertonic Saline

	**Mannitol**	**Hypertonic saline**
41. How are these agents administered?	☐ N/A<br>☐ Dose titrated to ICP<br>☐ Fixed Bolus dosing<br>☐ Standard continuous infusion	☐ N/A<br>☐ Dose titrated to ICP<br>☐ Fixed Bolus dosing<br>☐ Standard continuous infusion
42. If administered in fixed bolus doses, how frequently is it given:	☐ N/A<br>☐ More often than 6 times per day<br>☐ 6 times per day (every 4 hours)	☐ N/A<br>☐ More often than 6 times per day<br>☐ 6 times per day (every 4 hours)

	☐ 4 times per day (every 6 hours)<br>☐ Less than 4 times per day<br>☐ Other, please specify…………….	☐ 4 times per day (every 6 hours)<br>☐ Less than 4 times per day<br>☐ Other, please specify…………………….
	If mannitol is administered, is serum osmolarity monitored?<br><br>☐ N/A<br>☐ No<br>☐ Yes<br><br>If yes, an upper limit of ……….. mOsm/liter	If hypertonic saline is administered as a continuous infusion, is there a serum sodium goal?<br>☐ N/A<br>☐ No<br>☐ Yes<br><br>If yes, a goal of ………………….(mEq/L)
The responses to this question should represent, as best as practicable, a general consensus on treatment at your centre, rather than individual management preferences.		

Second/third tier therapies for treatment of raised intracranial pressure

The responses to the following questions should represent, as best as practicable, a general consensus on treatment at your centre, rather than individual management preferences.

43. Are the following approaches used to treat <u>refractory</u> intracranial hypertension?

	Never (0-10%)	Rarely (10-30%)	Sometimes (30-70%)	Frequently (70-90%)	Always (90-100%)
Decompressive craniectomy	☐	☐	☐	☐	☐
Hypothermia (temperature < 36 degrees Celsius)	☐	☐	☐	☐	☐
Intensive hyperventilation (PCO2 < 4.0 kPa)	☐	☐	☐	☐	☐
Barbiturates	☐	☐	☐	☐	☐

43b. If hyperventilation is answered with rarely – always:
When is hyperventilation (PaCO2 < 4,0 kpa)) utilized in Traumatic Brain Injury (TBI) patients at the intensive care unit?

	Never (0-10%)	Rarely (10-30%)	Sometimes (30-70%)	Frequently (70-90%)	Always (90-100%)
prophylactic hyperventilation (PaCO2 < 35 mmHg)	☐	☐	☐	☐	☐
To manage intracranial pressure for less than six hours	☐	☐	☐	☐	☐
To manage intracranial pressure for more than six hours	☐	☐	☐	☐	☐
In cases of imminent herniation	☐	☐	☐	☐	☐
Is brain tissue oxygen monitoring (PbtO2) used to measure cerebral oxygenation during hyperventilation?	☐	☐	☐	☐	☐

43c. If hyperventilation is used, what is the target PaCO2 (as second/third tier therapy)?

- o <35 mmHg
- o <30 mmHg
- o <25 mmHg

- Variable, dependent on patient
- Variable, dependent on surgeon/intensivist

43d. If hypothermia is used, what is the target temperature?
- >35°
- 35°
- 33 or 34°
- 32°
- Variable depending on patient
- Variable depending on physician

43e. If barbiturates are used, how is the dose targeted?
Select all that apply
- ☐ Continuous EEG monitoring
- ☐ Intermittent EEG recording
- ☐ Serum levels
- ☐ EEG and serum levels
- ☐ ICP control
- ☐ ICP control and EEG

Seizure prophylaxis and management

The responses to the following questions should represent, as best as practicable, a general consensus on treatment at your centre, rather than individual management preferences. These questions refer to the use of drugs to prevent seizures, rather than treat documented seizures

44. What are indications for anti-seizure prophylaxis in your centre?

	Never (0-10%)	Rarely (10-30%)	Sometimes (30-70%)	Frequently (70-90%)	Always (90-100%)
Glasgow Coma Scale (GCS) < 10	☐	☐	☐	☐	☐
Cortical contusion	☐	☐	☐	☐	☐
Depressed skull fracture	☐	☐	☐	☐	☐
Subdural hematoma	☐	☐	☐	☐	☐
Epidural hematoma	☐	☐	☐	☐	☐
Intracerebral hematoma	☐	☐	☐	☐	☐
Penetrating brain injury	☐	☐	☐	☐	☐
Other – please specify ……………………………….. …………………………………	☐	☐	☐	☐	☐

45. What is the duration of recommended anti-seizure <u>prophylaxis</u> at your hospital?
Select all that apply
- ☐ 1-3 days

- ☐ 4-7 days
- ☐ >7 days
- ☐ 3 weeks
- ☐ 3 months
- ☐ Variable depending on patient
- ☐ Variable depending on physician

46. Please rate the utilization of following agents used for seizure prophylaxis of Traumatic Brain Injury (TBI) patients:

	Always (90-100%)	Frequently (90-70%)	Sometimes (30-70%)	Rarely (10-30%)	Never (0-10%)
Phenytoin	☐	☐	☐	☐	☐
Levetiracetam	☐	☐	☐	☐	☐
Valproate	☐	☐	☐	☐	☐
Other, please specify………………..	☐	☐	☐	☐	☐

Treatment of seizures

The responses to the following questions should represent, as best as practicable, a general consensus on treatment at your centre, rather than individual management preferences.

47. Does your Intensive Care Unit (ICU) initiate anti-epileptic <u>treatment</u> after:

	Never (0-10%)	Rarely (10-30%)	Sometimes (30-70%)	Frequently (70-90%)	Always (90-100%)
A single seizure	☐	☐	☐	☐	☐
Two or more seizures	☐	☐	☐	☐	☐

48. Please rate your utilization of following agents used for seizure treatment of Traumatic Brain Injury (TBI) patients:

	Never (0-10%)	Rarely (10-30%)	Sometimes (30-70%)	Frequently (70-90%)	Always (90-100%)
Phenytoin	☐	☐	☐	☐	☐
Levetiracetam	☐	☐	☐	☐	☐
Valproate	☐	☐	☐	☐	☐
Other, please specify………………..	☐	☐	☐	☐	☐

Fever

The responses to the following questions should represent, as best as practicable, a general consensus on treatment at your centre, rather than individual management preferences.

49. Is fever (core temperature >38) in Traumatic Brain Injury (TBI) patients routinely treated in your unit?

- ○ Never (0-10%)
- ○ Rarely (10-30%)
- ○ Sometimes (30-70%)
- ○ Frequently (70-90%)
- ○ Always (90-100%)

50. Please rate your utilization of the following approaches for management of fever in Traumatic Brain Injury (TBI) patients with increased ICP:

	Never (0-10%)	Rarely (10-30%)	Sometimes (30-70%)	Frequently (70-90%)	Always (90-100%)
Paracetamol	☐	☐	☐	☐	☐
NSAIDs	☐	☐	☐	☐	☐
External cooling (cold blankets, etc)	☐	☐	☐	☐	☐
Intravascular cooling	☐	☐	☐	☐	☐

	Never (0-10%)	Rarely (10-30%)	Sometimes (30-70%)	Frequently (70-90%)	Always (90-100%)
51. Are corticosteroids used for the primary management of TBI in patients with head injury?	☐	☐	☐	☐	☐
52. Are corticosteroids used for ICU management of other conditions in TBI patients (e.g.: vasopressor resistant hypotension)? Specify: ………………………………..	☐	☐	☐	☐	☐

Deep venous thrombosis (DVT) prophylaxis

The responses to the following questions should represent, as best as practicable, a general consensus on treatment at your centre, rather than individual management preferences.

	Never (0-10%)	Rarely (10-30%)	Sometimes (30-70%)	Frequently (70-90%)	Always (90-100%)
53. How often is	☐	☐	☐	☐	☐

DVT prophylaxis used?

54. If you use DVT prophylaxis, when is DVT prophylaxis initiated?

	< 24 hrs	24-72 hrs	< 72 hrs	Never
In the absence of hemorrhagic lesions	☐	☐	☐	☐
In the presence of hemorrhagic lesion	☐	☐	☐	☐
After intracranial surgery	☐	☐	☐	☐

55. In patients who receive DVT prophylaxis, what medication is given?

- ☐ Subcutaneous unfractioned heparin
- ☐ Low-molecular weight heparin
- ☐ Other, please specify…………………………………………………………………

56. Coagulopathy related to the trauma is treated with :

	Never (0-10%)	Rarely (10-30%)	Sometimes (30-70%)	Frequently (70-90%)	Always (90-100%)
Fresh Frozen plasma (FFP)	☐	☐	☐	☐	☐
Platelets	☐	☐	☐	☐	☐
Fibrinogen	☐	☐	☐	☐	☐
Novo 7 (recombinant factor VII)	☐	☐	☐	☐	☐
Vitamin K	☐	☐	☐	☐	☐
PCC (Prothrombin Complex Concentrate)	☐	☐	☐	☐	☐
Other, please specify…	☐	☐	☐	☐	☐

Respiration and ventilation

The responses to the following questions should represent, as best as practicable, a general consensus on treatment at your centre, rather than individual management preferences.

Mechanical ventilation

57. Select initial PaO2 goal in mechanically ventilated Traumatic Brain Injury (TBI) patients:

- o > 8 kpa (60 mmHg)
- o > 10 kpha (75mmHg)

- ○ >13 kpa (100 mmHg)
- ○ Other, please specify……………………

58. Select initial arterial oxygen saturation goal in mechanically ventilated Traumatic Brain Injury (TBI) patients:

- ○ > 85%
- ○ > 90 %
- ○ > 95%
- ○ Other, please specify……………………………

59. In patients remaining unconscious after Traumatic Brain Injury (TBI) , when would your ICU consider a tracheostomy?

- ○ Within 1 week
- ○ 1- 2 weeks
- ○ > 2weeks

60. Select PaCO2 goal for management in mechanically ventilated Traumatic Brain Injury (TBI) patients:

	25-29 mmHg	30-35 mmHg	36-40 mmHg	41-45 mmHg
In the absence of (suspicion of) raised ICP	○	○	○	○
In the presence of raised ICP	○	○	○	○

General ICU treatments / protocols

Red blood cell policy

61. Does the Intensive Care Unit (ICU) protocol specify a target goal for hemoglobin concentration?

- ○ No
- ○ Yes, please specify …………………………………………..

62. Do you have a transfusion target in patients with Traumatic Brain Injury (TBI) in the acute phase?

- ○ >100 g/l or 6 mmol/l
- ○ Between 90g/l or 5.5 mmol/l and 100 g/l or 6 mmol/l
- ○ between 80 g/l or 5 mmol/l and 90 g/l or 5.5 mmol/l
- ○ Between 70 g/l or 4.0 mmol/l and 80 g/l or 5 mmol/l

63. What is your transfusion target in patients with non-neurological critical illness?

- ○ >100 g/l or 6 mmol/l
- ○ between 90g/l or 5.5 mmol/l and 100 g/l or 6 mmol/l
- ○ between 80 g/l or 5 mmol/l and 90 g/l or 5.5 mmol/l
- ○ Between 70 g/l or 4.0 mmol/l and 80 g/l or 5 mmol/l

Glucose

64. Is there a standard protocol for glucose management in Traumatic Brain Injury (TBI) in your Intensive Care Unit (ICU) ?

- ○ No
- ○ Yes

65. What therapy is used in glucose management at your Intensive Care Unit (ICU) ?

- ☐ No specific therapy

- ☐ Prophylactic insulin administration (buffered infusion)
- ☐ Insulin administration to correct hyperglycemias
- ☐ Tight glycemic control

Caloric intake / nutrition

66. How is nutrition managed at your Intensive Care Unit (ICU) ?
    - o Always parenteral route
    - o Always Enteral route
    - o Mostly parenteral route, enteral route on indication
    - o Mostly enteral route, parenteral route on indication
    - o Other……………………………………….

67. What caloric intake do you aim for in patients with Traumatic Brain Injury (TBI) at your Intensive Care Unit (ICU) ?

……………………… Kcal/kg/day
- ☐ Unknown / no protocol

68. When do you usually start parenteral nutrition at your Intensive Care Unit (ICU) in Traumatic Brain Injury (TBI) patients?
    - o As soon as possible (directly after ICU admission)
    - o Within 24 hours post-injury
    - o Within 72 hours post-injury
    - o Within 7 days post-injury
    - o We do not have rules / guidelines for this

69. When do you usually aim for full caloric intake replacement?
    - o At 7 days post-injury
    - o < 7 days post-injury
    - o > 7 days post-injury

Outcome

70. Does your hospital routinely assess the outcome at follow-up according to the Glasgow Outcome Scale (extended)?
    - o No
    - o Yes
    - o Unknown

70b. If yes: Is a structural interview used to assess the Glasgow Outcome Scale (extended)?
- o No
- o Yes
- o Unknown

70c. If yes: Who usually assesses the GOS(E)?
Select all that apply
- ☐ Research nurse
- ☐ Nurse
- ☐ Clinician
- ☐ Other, please specify….

71. Which of the following reasons for disability does your hospital include or exclude in your assessment of the GOS(E)?

In the responses below, you can choose either “include” or “Exclude” as a response to each item. If you tick “Include” means that you assess all of the disability as part of categorizing the patient on the GOSE. If you tick “Exclude” this means that where the disability is thought to be the consequence of an injury other than TBI, it will not be included in the assessment, and you would assign the GOS(E) as if that disability did not exist.

	Include	Exclude
Effects of health conditions that existed before the injury, such as cognitive impairment or physical limitations.	☐	☐
Effects of injuries sustained on the same occasion to parts of the body other than the head, such as paralysis due to spinal cord injury or injuries to the limbs.	☐	☐
Effects of external damage to the head or injury to the skull, such as limitations in activities due to a missing bone flap.	☐	☐
Effects of illness arising after TBI treatment, such as pulmonary complications after ventilation.	☐	☐
Effects of a subsequent illness unconnected to TBI, such as pneumonia after flu.	☐	☐
Effects of a subsequent operation, such as hip replacement, that is unconnected to TBI.	☐	☐
Effects of changed social circumstances, such as lower income after injury.	☐	☐
Effects of depression that has arisen since the TBI.	☐	☐
Effects of post-traumatic stress disorder that has appeared since the TBI.	☐	☐
Effects of post-injury anxiety states, such as the development of agoraphobia.	☐	☐

Delirium

72. With regards to the assessment of delirium, does your ICU undertake:

- o No assessment of delirium
- o Assessment of delirium based on general clinical assessment
- o Assessment of delirium using an ICU scale for delirium, such as the CAM-ICU, ICDSC, DDS, Nu-DESC

The DSM criteria for delirium include a disturbance of consciousness with impaired attention; a change in cognitive function (eg, memory impairment, disorientation, or language disturbance) or a perceptual disturbance; the disturbance develops over a short period of time (hours to days) and fluctuates; and the history, physical examination, or laboratory data suggest that the abnormalities are caused by a general medical condition and are not better accounted for by a preexisting dementia. Delirious patients are classified into two behavioral subtypes: ‘‘hyperactive delirium,’’ in which patients are agitated, loud, combative, and likely to inflict significant harm on themselves or others, and ‘‘hypoactive delirium,’’ in which they are withdrawn and have minimal interaction with health providers or family.

Provider Profiling Questionnaire

Questionnaire 8: Ethical aspects of the ICU

This questionnaire can be completed by a(n) (neuro)intensivist in collaboration with a neurosurgeon / trauma surgeon

This questionnaire includes questions about the general policy in your hospital. The responses to these questions should represent, as best as practicable, a general consensus on treatment at your centre, rather than individual management preferences. Consequently, you should provide responses that describe not what you would do personally, but how the majority of patients would generally be treated in your centre.

There are no 'right' or 'wrong' answers so please give us a realistic and honest view of how the care in your hospital is organized. Your answers will only be used to answer the scientific questions in CENTER TBI and no information in any form will be reported on individual centre level. Some of the questions may seem similar, but please answer all questions.

If you have any questions or problem, please contact:
Maryse Cnossen, PhD student (m.c.cnossen@erasmusmc.nl)

Information about the completer of the questionnaire

Other than the CENTER-TBI investigator, which of the following individuals was involved in completion of this questionnaire?
Select all that apply

- ☐ Neurologist
- ☐ Neurosurgeon
- ☐ Trauma Surgeon
- ☐ Emergency Department (ED) physician
- ☐ Administrative staff member / data manager / financial department
- ☐ Other, please specify……………..
- ☐ NA. The questionnaire was completed solely by the CENTER TBI local investigator

The Local investigator is the senior clinician(s) at your hospital involved in supervision of CENTER TBI

Withdrawal of life support

1.Of all patients with severe neurological damage who die on the ICU, approximately, how many die after withdrawal of life-sustaining measures?

- o 0-25%
- o 26-50%
- o 51-75%
- o >75%

2. How is the decision reached to withhold/withdraw life-sustaining measures (e.g. mechanical ventilation, vasoactive medication, renal replacement therapy, intravenous fluid administration)?
Select all that apply

- ☐ One physician (e.g. the most senior person) decides following multidisciplinary discussion
- ☐ During multidisciplinary discussion in which the majority (more than 50%) has to agree
- ☐ During multidisciplinary discussion in which there has to be unanimous consensus among all participating doctors
- ☐ One physician decides (along with objective medical criteria) without multidisciplinary discussion (veto)
- ☐ Other, please specify …………………………….

3. How is a decision reached to not treat patients surgically, because the primary brain damage is considered too devastating (poor prognosis)?
Select all that apply

- ☐ One physician (e.g. the most senior person) decides following multidisciplinary discussion
- ☐ During multidisciplinary discussion in which the majority (more than 50%) has to agree
- ☐ During multidisciplinary discussion in which there has to be unanimous consensus among all participating doctors
- ☐ One physician decides (along with objective medical criteria) without multidisciplinary discussion (veto)
- ☐ Other, please specify …………………………….

4. To what extent do opinions of legal representatives/relatives play a role in decision-making about withdrawal/withholding of life-sustaining measures?

- o None, treatment decisions are always made by the medical staff
- o Treatment decisions are always made by medical staff but legal representatives/relatives can ask for a second opinion

- To some extent
- To a great extent, their opinions are leading in the decision making

5. If the decision is made to withdraw life-sustaining measures, do you preemptively administer sedatives and opioids?
    - No
    - Yes
    - Sometimes
    - Unknown

6. If the decision is made to withdraw life-sustaining measures and before actual withdrawal, do you initiate palliative therapy in anticipation of distressing symptoms (such as pain, terminal restlessness, death rattle, stridor, dyspnoea)?

- No, we withdraw all treatment, including administration of sedatives and/or opioids, and treat symptoms only if they occur
- Yes, we withdraw all treatment, but we provide anticipatory treatment of distressing symptoms only with continuation of sedatives and/or opioids in doses which were given before withdrawal
- Yes, we withdraw all treatment, but we provide anticipatory therapy only with continuation and subsequent increase of sedatives and/or opioids beyond (=dosed higher than strictly needed for) comfort
- Yes, we withdraw all treatment, but we provide anticipatory treatment with continuation of sedatives and opioids and give anticipated medication for stridor and death rattle and other distressing symptoms
- Yes, we withdraw all treatment, but we provide anticipatory treatment with continuation of sedatives and opioids and subsequent increase of sedatives and/or opioids beyond (=dosed higher than strictly needed for) comfort and give anticipated medication for stridor and death rattle and other distressing symptoms
- Other, please specify ………………………..

6b. If yes: is this formalized in a protocol?
- No
- Yes

7. If the decision is made to withdraw life-sustaining measures, do you remove the endotracheal tube?
- No, never
- Yes, always
- Sometimes
- Only on request of the relatives

8. If the decision is made to withdraw life-sustaining measures in a comatose severely injured TBI patent, which life-sustaining measures do you stop?
Select all that apply
- ☐ We stop mechanical ventilation
- ☐ We stop administration of vasoactive medication
- ☐ We stop renal replacement therapy
- ☐ We stop administration of intravenous fluids

- We stop nasogastric feeding

9. If the decision is made to withdraw life-sustaining measures, in a patient with high intracranial pressure, but who is not brain dead, would you remove the ventricular drain (for CSF drainage), but continue other life-sustaining measures in the hope that the patient will become brain dead and thereby becomes a suitable candidate for organ donation?
   - No, never
   - Yes, sometimes
   - Yes, always

10. If the patient has a GCS of 3 and non-reactive pupils and he/she is NOT a candidate for organ donation, do you:

    Test brainstem reflexes (oculocephalic reflexes, oculovestibular reflexes, corneal reflexes, cough reflex) before withdrawal of mechanical ventilation?
    - No
    - Yes

    When answered 'yes' do you withdraw ventilation only if the brainstem reflexes are negative?
    - No
    - Yes

    Order ancillary tests, as electroencephalogram, transcranial Doppler, CT-angiography?
    - No
    - Yes

    When answered 'yes' do you withdraw ventilation only if the ancillary tests are negative?
    - No
    - Yes

    Test apnea?
    - No
    - Yes

11. When do you declare a patient brain dead?
    - With GCS 3, fixed dilated pupils and no confounding factors [e.g. hypothermia, barbiturates]
    - With GCS 3 and absent brain stem reflexes and no confounding factors
    - With GCS 3, absent brain stem reflexes and apnea and no confounding factors
    - With GCS 3, absent brain stem reflexes, apnea and ancillary test(s) (eg EEG or cerebral angiography) and absence of confounding factors
    - Other, please specify …………………………………….

12. Must the patient, who is not suitable for organ donation, be declared brain dead before withdrawal of life-sustaining measures?
    Select all that apply
    - No, the prospect of a very poor prognosis can be enough
    - No, GCS 3 and fixed dilated pupils and no confounders is enough to stop treatment
    - Yes, this is mandatory by law in my country
    - Yes, it is not mandatory by law, but I always do that to be sure

13. Would you consider organ donation after circulatory arrest in a patient in whom mechanical ventilation will be withdrawn, but who is not brain dead?
    - No, this is forbidden in my country

- No, although it would be permitted, I would not do this
- Yes, sometimes
- Yes, always

14. After withdrawal of mechanical ventilation and after circulatory arrest, when exactly do you declare the patient dead in case of a circulatory death organ donor?
    - directly after circulatory arrest determined after a 'flatliner-ECG' on the monitor
    - after one minute 'flatliner-ECG' indicating circulatory arrest
    - after two minutes 'flatliner-ECG'
    - after five minutes 'flatliner-ECG'
    - after ten minutes 'flatliner-ECG'
    - after loss of pulsatile arterial curve on the invasive arterial blood pressure tracing
    - Other, please specify …………………………………….

15. After withdrawal of mechanical ventilation and after circulatory arrest, after how many minutes circulatory arrest do you declare the patient dead in cases not suitable as organ donor ?
    - directly after circulatory arrest determined after a 'flatliner-ECG' on the monitor
    - after one minute 'flatliner-ECG'
    - after two minutes 'flatliner-ECG'
    - after five minutes 'flatliner-ECG'
    - after ten minutes 'flatliner-ECG'
    - after loss of pulsatile arterial curve on the invasive arterial blood pressure tracing
    - Other, please specify ……………………………….

16. At what time after injury would you consider to withdraw life support in a patient with TBI, who is in a very poor prognostic condition (based on CT scan, GCS, clinic, ICP etc), but not brain dead?

	Never	Sometimes	Often	Always
24 hours	☐	☐	☐	☐
2-4 days	☐	☐	☐	☐
4-7 days	☐	☐	☐	☐
>1week	☐	☐	☐	☐
>2weeks	☐	☐	☐	☐

17. Does the age of the patient influence your decision making about withholding and withdrawing life-sustaining treatment?

    - Yes, always, independent of other criteria
    - Yes, but only in combination with other criteria as CT scan, GCS, depth of coma
    - No, I only decide on the severity of the injury and anticipated prognosis

Age and ICU admission

18. Do you admit very elderly (80 years and older) patients with severe TBI on the ICU for treatment?

    - No, never
    - Yes, if the patient is intubated and ventilated in the ER setting
    - Yes, if the patient needs ICU treatment with the prospect of saving his/her life
    - Yes, but highly depending on the severity of co-morbidity
    - Yes, but only if the relatives ask me

19. What are possible reasons for admitting a very elderly patient with severe TBI to the ICU?

Select all that apply

- ☐ Age is not a criterion for triage
- ☐ Elderly patients can benefit from ICU admission
- ☐ Prognosis is unsure
- ☐ Good pre-injury quality of life
- ☐ Absence of pre-injury level of frailty and disability
- ☐ Absence of pre-injury co-morbidity
- ☐ Everybody, independent of age, deserves a chance

20. What are possible reasons for *not* admitting a very elderly patient with severe TBI to the ICU?

Select all that apply

- ☐ The patient is 80+
- ☐ Patients or family do not want any escalation of care
- ☐ Anticipated prognosis
- ☐ These patients are more prone to severe iatrogenic complications from invasive measures and monitoring
- ☐ Impaired pre-injury quality of life
- ☐ Pre-injury level of frailty and disability
- ☐ Pre-injury co-morbidity
- ☐ Scarcity of ICU beds
